# Complete chloroplast genomes from apomictic *Taraxacum* (Asteraceae): Identity and variation between three microspecies

**DOI:** 10.1371/journal.pone.0168008

**Published:** 2017-02-09

**Authors:** Rubar Hussein M. Salih, Ľuboš Majeský, Trude Schwarzacher, Richard Gornall, Pat Heslop-Harrison

**Affiliations:** 1 Department of Genetics, University of Leicester, Leicester, United Kingdom; 2 Field crops department, Faculty of Agricultural Sciences, University of Sulaimani, Sulaimani, Kurdistan Region, Iraq; 3 Department of Botany, Faculty of Science, Palacky University in Olomouc, Olomouc, Olomouc-Holice, Czech Republic; Austrian Federal Research Centre for Forests BFW, AUSTRIA

## Abstract

Chloroplast DNA sequences show substantial variation between higher plant species, and less variation within species, so are typically excellent markers to investigate evolutionary, population and genetic relationships and phylogenies. We sequenced the plastomes of *Taraxacum obtusifrons* Markl. (O978); *T*. *stridulum* Trávniček ined. (S3); and *T*. *amplum* Markl. (A978), three apomictic triploid (2n = 3x = 24) dandelions from the *T*. *officinale* agg. We aimed to characterize the variation in plastomes, define relationships and correlations with the apomictic microspecies status, and refine placement of the microspecies in the evolutionary or phylogenetic context of the Asteraceae. The chloroplast genomes of accessions O978 and S3 were identical and 151,322 bp long (where the nuclear genes are known to show variation), while A978 was 151,349 bp long. All three genomes contained 135 unique genes, with an additional copy of the *trn*F*-*GGA gene in the LSC region and 20 duplicated genes in the IR region, along with short repeats, the typical major Inverted Repeats (IR1 and IR2, 24,431bp long), and Large and Small Single Copy regions (LSC 83,889bp and SSC 18,571bp in O978). Between the two *Taraxacum* plastomes types, we identified 28 SNPs. The distribution of polymorphisms suggests some parts of the *Taraxacum* plastome are evolving at a slower rate. There was a hemi-nested inversion in the LSC region that is common to Asteraceae, and an SSC inversion from *ndh*F to *rps*15 found only in some Asteraceae lineages. A comparative repeat analysis showed variation between *Taraxacum* and the phylogenetically close genus *Lactuca*, with many more direct repeats of 40bp or more in *Lactuca* (1% larger plastome than *Taraxacum*). When individual genes and non-coding regions were for Asteraceae phylogeny reconstruction, not all showed the same evolutionary scenario suggesting care is needed for interpretation of relationships if a limited number of markers are used. Studying genotypic diversity in plastomes is important to characterize the nature of evolutionary processes in nuclear and cytoplasmic genomes with the different selection pressures, population structures and breeding systems.

## Introduction

The organization of chloroplast genomes (plastomes) has similarities at the structural and gene level across higher plants [1, 2]. The DNA sequences show characteristic variation depending on their taxonomic position, and sequence fragments are widely exploited in molecular taxonomy [3]. The chloroplast (or, more generally, plastid) genome (plastome, ctDNA, cpDNA) shows maternal inheritance in most species [4] and normally there is only one haplotype in a plant. Since there is no sexual recombination among plastomes (although horizontal transfer of whole chloroplasts [5], or chloroplast capture [6] may occur), chloroplast markers can give robust phylogenies and are then used to estimate divergence times between lineages [7]. The sequencing of the first plastome in *Nicotiana tabacum* [8] has been followed by some 626 chloroplast whole plastomes belonging to 133 different plant families (including 18 well-defined species from 16 genera in the Asteraceae) deposited in the NCBI Organelle Genome Resources database by early 2016 [9–11].

Typical angiosperm plastome sizes range from 135 to 160 kb (although much reduced in hemi-parasitic plants). The plastome has a conserved quadripartite structure composed of two copies (ca. 25 kb) of an Inverted Repeat (IR) which divides the remainder of the plastome into one Large and one Small Single Copy region (LSC and SSC) [1, 2]. One monophyletic clade within the legumes (including the tribes Cicereae, Hedysareae, Trifolieae and Fabeae and some other genera; see Wojciechowski [12]) and all conifers [13] have smaller plastomes in which one copy of the inverted repeat is missing, defining evolutionary lineages. Using whole plastid sequences from two orchid species, Luo *et al*. [14] demonstrated that chloroplast structure, gene order and content are similar but differ with expansions and contractions at the inverted repeat-small single-copy junction and *ndh* genes.

PCR-amplified sequences within plastomes are used extensively for species identification and reconstruction of phylogeny at around the species level. Several regions are consistently the most variable across angiosperm lineages and some are widely used for barcoding approaches for purposes such as species discovery, floristic surveys, identification of plants, or identification of composition of natural products (e.g. Bruni *et al*. [15]; Bruni *et al*. [16]; Hollingsworth *et al*. [17]), following amplification and sequencing: *ndh*F*-rpl*32, *rpl*32*-trn*L*-*UAG, *ndh*C*-trn*V, 5'*rps*16*-trn*Q, *psb*E*-pet*L, *trn*T*-psb*D, *pet*A*-psb*J, and *rpl*16 (e.g. Dong *et al*. [18]). However, there is no universal ‘best’ region. The average number of regions applied to inter specific studies is about 2.5 which may be too little to access the full discriminating power of this plastome [19]. It is important to have multiple complete plastome for species across a family as references both to characterize any major structural changes, which would be difficult to identify from fragments, and to aid design of conserved PCR primers to exploit polymorphic regions in larger samples within and between taxa.

What are the limits on use of chloroplast sequences for addressing taxonomic questions? The answer depends on the rates of evolution and nature of variation found at different regions of the plastome. Shaw *et al*. [19] commented on the use of plastome sequences at increasingly low taxonomic levels: the genes most commonly analysed after amplification by PCR may be appropriate for delineation of species but may not represent the most variable regions of the chloroplast. In date palm, chloroplast haplotypes may correlate with populations [20], although founder effects may be strong in such species. Särkinen and George [21] used full plastome sequences of *Solanum* chloroplasts to identify the most variable plastid markers, concluding that different chloroplast regions are appropriate for study of evolution at different taxonomic levels from family downwards.

In the Asteraceae, Wang *et al*. [22] have analysed 81 genes from chloroplasts of 70 different species, showing the family is monophyletic and branching is consistent with tribal relationships as understood on the basis of morphology. The Asteraceae family includes an inversion in the plastome relative to other eudicots [23]. The boundaries of a 22.8 kb inversion define a split within the family, and a second 3.3 kb inversion is nested within the larger inversion. Generally, one of the end points of the smaller inversion is upstream of the gene *trn*E, and the other end point is located between the gene *trn*C and *rpo*B. The two inversions are similar among members of the Asteraceae lineage suggesting that the second inversion event occurred within a short evolutionary time after the first event. Estimates of divergence times based on *ndh*F and *rbc*L gene sequences suggest that two inversions originated during the late Eocene (38–42 MYA), soon after the Asteraceae originated in the mid Eocene (42–47 MYA) [23].

The genus *Taraxacum* (Cichorieae, Asteraceae) is known for its complex reticular evolution including polyploidy events, hybridization and apomixis [24] that makes it difficult to reconstruct a reliable phylogeny. Repeated hybridization between sexual (diploids or rarely tetraploids) and apomictic (triploids and higher ploidies) taxa, rapid colonization of wide areas by apomicts after the Last Glacial Maximum (LGM), low levels of morphological differentiation and remaining ancestral sequence polymorphisms have been of interest and a challenge to botanists for more than a century (e.g. Nägeli, having seen the results of Mendel [25], suggested that Mendel should investigate the apomictic *Hieracium* species, see [26–30]). Investigation of genotypic diversity in pure apomictic and mixed sexual-apomictic populations showed variation arises from both mutation (accumulation of somatic mutations/allele divergence) and recombination (gene flow between sexual-apomictic individuals) [31–34]. Utilization of common chloroplast markers from coding and non-coding regions showed at best weak differentiation within the genus but helped to distinguish evolutionary old and primitive from evolutionary younger or more advanced groups of haplotypes [35, 36]. Nevertheless, observed haplotypes were not species specific, some being rare while others were frequent and shared among different and not related taxa, even between sexual and apomictic plants (e.g. [32, 34, 35]). Mes *et al*. [37] showed a high level of homoplasy in several non-coding plastome regions.

Here we aimed to sequence whole chloroplast genomes (plastomes) of three morphologically well-defined apomictic microspecies or agamospecies from the *Taraxacum officinale* aggregate (dandelions), namely *T*. *obtusifrons*, *T*. *stridulum* and *T*. *amplum*. Our goals were to characterize the nature and scale of differentiation between plastomes in three related apomictic taxa and see if there were features of plastome variation that may be a consequence of apomixis. We then aimed to find the evolutionary relationships between the plastomes in the microspecies, and place them phylogenetically in the genus *Taraxacum*, the tribe Cichorieae and the Asteraceae. The results also aimed to identify appropriate regions for use as markers in future studies comparing mutation and inheritance of the nuclear genome in the apomicts with the maternally inherited plastome.

## Materials and methods

### Plant material and DNA sequencing

Three agamospecies (2n = 3x = 24) of *Taraxacum officinale* agg. [section *Taraxacum* (formerly *Ruderalia*), Asteraceae], *T*. *obtusifrons* Markl. (O978); *T*. *stridulum* Trávniček ined. (S3); and *T*. *amplum* Markl. (A978) were germinated and planted in pots. The seeds came from the agamospermous progeny of maternal plants genotyped by nuclear markers by Majeský *et al*. [32] and ploidy was measured by chromosome counts and flow cytometry [32]. Geographical records of origin and voucher specimens are deposited in the Herbarium of the Department of Botany, Palacký University, Olomouc, Czech Republic (herbarium abbreviation: OL). Nuclear markers confirm the genotypes used for sequencing; plants were karyotyped showing 2n = 3x = 24 chromosomes, and voucher specimens of the sequenced plants have been deposited in the University of Leicester, UK, herbarium (LTR). Total DNA including nuclear, mitochondrial and plastome DNA was extracted from fresh green young leaves using standard cetyl-trimethyl-ammonium bromide (CTAB) methods [38] to obtain high quality DNA.

DNA was sequenced commercially (Interdisciplinary Center for Biotechnology Research, University of Florida, USA); accession S3 was sequenced with Illumina Miseq 2x300bp paired end reads while accessions O978 and A978 were sequenced using Illumina Hiseq500 2x150bp reads. About 59,258,642 paired-end reads were obtained for S3 (22 Gb), and 58,713,854 and 69,056,774 paired-end reads (12 Gb) were obtained for A978 and O978 respectively.

### Sequence assembly

Assembly and analysis of the plastomes were performed on Ubuntu Linux 13.10, with Geneious version 7.1.4 and later [39] (available from http://www.geneious.com/). Using paired end reads from S3, *de novo* assembly generated one large contig of >150,000 bp (420,584 reads) which was largely homologous to the *Lactuca sativa* var. *salinas* (DQ_383816; Asteraceae) [40] plastome which was then used to generate a consensus reference sequence. For A978 and O978, and for final assembly of the S3 plastome, all raw reads were mapped to the S3 reference (five iterations). The initial assembly showed some areas of double-coverage of repeated regions, and minimal coverage at the four junctions between IRs and the SSC/LSC regions; repeated assembly to short regions corrected these, until uniform coverage with no assembly gaps, high similarity of all assembled reads to the consensus, and minimal unmatched paired reads, was achieved. Plastome bases were numbered so the first base pair after IR2, immediately before the *trn*H gene, became base number 1.

### Plastome annotation

Coding sequences and directions were identified in the *Taraxacum* plastome and genes; rRNA and tRNAs were annotated with the Geneious annotation function and DOGMA (Dual Organellar Genome Annotator [41], http://bugmaster.jgi-psf.org/dogma/) with reference to published plastomes. In particular, the *Taraxacum* annotation was optimized by comparison with *Lactuca* (DQ_383816) to identify gene and exon boundaries, and tRNA genes were further confirmed with the online tRNAscan-SE 1.21 search server [42]. A circular plastome map was drawn using the online program GenomeVX [43].

### Short repeat motifs

REPuter [44] was used to identify and locate DNA repeats including direct (forward), inverted (palindrome) repeats, reverse, and complementary sequences more than 20 bp long (90% identity; Hamming distance 2). TandemRepeatFinder [45] was used to find tandem repeats.

### Comparison of chloroplast features and phylogenetic analyses

To see the extent of difference between *Taraxacum* and 21 Asteraceae accessions with full plastome sequences, GC content, genome size, gene content and nature of LSC/SSC/IR were compared. Further, we compared the plastid sequences among 18 species and 16 genera in Asteraceae aligning the entire chloroplast (downloaded from GenBank) and the three *Taraxacum* plastomes. Based on primary alignment, regions with the highest sequence divergence were visualised in mVISTA program [46] in Shuffle-LAGAN mode with default parameters to reveal their sequence variation. The alignments were visually checked and edited manually. Based on the comparison of plastome sequences, the regions with highest sequence polymorphism levels were chosen for further phylogenetic analyses. The aim of the phylogenetic analyses was to examine the congruence of the phylogenetic trees with respect to placement of the three *Taraxacum* microspecies within the subsampled Asteraceae family (with the whole plastome sequences available) and with respect to used plastome region for phylogeny reconstruction.

Maximum Likelihood fits of 24 different nucleotide substitution models for 22 accessions using the whole chloroplast genome plus 40 genic and inter-genic regions were calculated, and evolutionary analyses were conducted in MEGA6 [47].

Phylogenetic analysis was conducted using the maximum likelihood (ML) method based on the best-fitted model of evolution as outlined in S1 Table. The bootstrap consensus tree was inferred from 1000 replicates [48]. Branches corresponding to partitions reproduced in less than 50% bootstrap replicates are collapsed. Initial tree(s) for the heuristic search were obtained automatically by applying Neighbor-Joining and BioNJ algorithms to a matrix of pairwise distances estimated using the Maximum Composite Likelihood (MCL) approach, and then selecting the topology with superior log likelihood value. A discrete Gamma distribution was used to model evolutionary rate differences among sites (4 categories). All three codon positions were included. Analyses were conducted in MEGA6 [47]. Trees were built for the entire plastome, 24 non-coding intergenic regions, 11 coding regions (including one intron), as well as separate analyses for the LSC, SSC and IR regions, tRNA and rRNA, genes in order to evaluate intragenomic variation in rates of molecular evolution, using *Nicotiana tabacum* (Solanaceae) as the outgroup.

## Results

### Structure of *Taraxacum* chloroplasts

**Fig 1:**
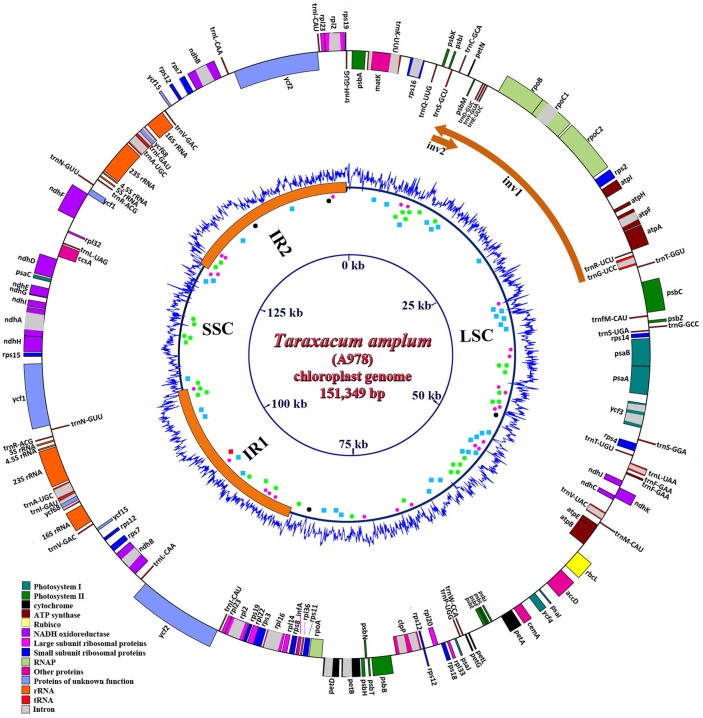
Map of the plastome of *Taraxacum amplum* (A978). Genes are shown inside or outside the circle to indicate clockwise or counterclockwise transcription direction respectively. The Inverted Repeat (IR, 24,431bp) is indicated by a thicker line for IR1 and IR2. GC content is show in the inner blue graph. Small Single Copy (SSC) and long single copy (LSC) regions are indicated, and the inverted regions (Inv1 and Inv2) within LSC relative to other species are shown as orange arcs. Short tandem repeats (microsatellites and minisatellites) are indicated by blue dots, palindromes by red dots, forward repeats by green dots and reverse repeats by black dots.

**Fig 2:**
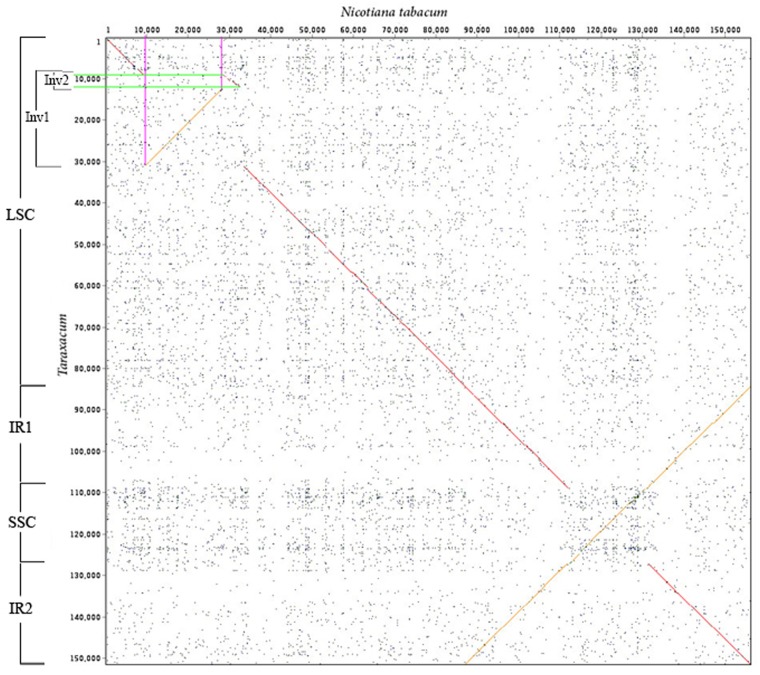
Dot-plot sequence comparison of *Taraxacum* and *Nicotiana* chloroplast sequences, showing the Inverted Repeats (IR1 and IR2), hemi-nested inversions between the two plastomes (Inv1 and Inv2) and inversion of the SSC.

Circular plastomes were assembled from the whole genome sequence data (average plastid coverage >2000 fold for each accession). The chloroplasts of accessions O978 and S3 were identical and 151,322 bp long, while A978 was 151,349 bp long. Fig 1 shows the circular map for the A978 accession, with genes, short repeats, the major Inverted Repeats (IR1 and IR2; 24,431 bp; see Fig 2), and LSC/SSC regions (LSC 83,889bp and SSC 18,571bp in O978 and S3). GC content (blue graph) was higher than average in the 7kb of the Inverted Repeat regions adjacent to the SSC.

### Chloroplast genome polymorphism between Taraxacum microspecies

Between the two *Taraxacum* plastomes, there were 28 SNPs (9 transversions and 19 transitions; Chi-square = 15.1; p = 0.0001), occurring in all regions of the plastome (13 in LSC, 13 in SSC and 2 in IRs; Table 1 and Fig 1). Two SNPs in LSC genes (*rpo*C1 and *acc*D) were non-synonymous changes with the other 9 SNPs in genes being synonymous. There were 16 indels between 1 and 24 bp long, all but one occurring within the LSC region (the LSC representing 55% of the plastome; p<0.001; Table 1). A unique 22bp insertion, the duplicated 11bp motif TGTAGACATAA in an intron of the *trn*L*-*UAA gene, was present in accession A978 (S1 Fig). Overall, non-coding regions show a higher sequence divergence than coding regions in *Taraxacum* (Table 1). In the sequence alignment, the highest divergence was seen in regions including the intergenic spacer of *trn*H*-psb*A, *trn*K*-rps*16, *rps*16*-trn*Q, *trn*S*-trn*C, *trn*C*-pet*N, *rpo*C2*-rps*2, *psb*Z*-trn*G, *trn*G*-trnf*M, *ycf*3*-trn*S, *trn*T*-trn*L, *trn*F*-ndh*J, *trn*M*-atp*E, *pet*B*-pet*D, *trn*N*-ycf*1, *ycf*1*-rps*15, *ndh*D*-ccs*A, *rpl*32*-ndh*F, *psbI-trnS*, *ndhF-ycf1* and *ndhI-ndhG*.

**Table 1:** Transition/transversion and insertion/deletion events between *Taraxacum* microspecies S3/O978 and A978; where indel occurs in a gene, the gene name is indicated; other indels are intergenic.

#	Type	Position	Location	Nucleotide position	S3/O978	A978
1	SNP	*LSC/trnK-rps16*	IGS*	4907	T	C
2	SNP	*LSC/rps16-trnQ*	IGS	6402	A	G
3	SNP	*LSC/trnS-trnC*	IGS	8856	A	G
4	SNP	*LSC/trnF-ndhJ*	IGS	47823	G	A
5	SNP	*LSC/ndhC-trnV*	IGS	50219	T	C
6	SNP	*LSC/psbB*	gene	72455	A	G
7	SNP	*LSC/rpl22*	gene	83275	T	C
8	SNP	*IR-1/ycf2-trnL*	IGS	93366	G	A
9	SNP	*SSC/ycf1*	gene	109145	A	G
10	SNP	*SSC/ycf1*	gene	111110	G	A
11	SNP	*SSC/ycf1*	gene	112536	T	C
12	SNP	*SSC/ycf1-rps15*	IGS	113190	A	G
13	SNP	*SSC/ndhD*	gene	120836	A	G
14	SNP	*SSC/ndhD-ccsA*	IGS	121274	A	G
15	SNP	*SSC/ndhD-ccsA*	IGS	121275	G	A
16	SNP	*SSC/rpl32-ndhF*	IGS	124080	T	C
17	SNP	*IR-2/trnL-ycf2*	IGS	141978	C	T
18	SNP	*LSC/trnH-psbA*	IGS	222	A	C
19	SNP	*LSC/trnS-trnC*	IGS	8715	T	G
20	SNP	*LSC/rpoC1*	gene	18257	A	C
21	SNP	*LSC/rpoC2*	gene	20210	A	C
22	SNP	*LSC/accD*	gene	57577	T	A
23	SNP	*LSC/psaL-ycf4*	IGS	59566	A	C
24	SNP	*SSC/psbB*	gene	72515	C	A26
25	SNP	*SSC/ycf1*	IGS	112416	C	G
26	SNP	*SSC/ndhF*	gene	126757	A	C
27	SNP	*SSC/ndhD-ccsA*	IGS	121278	A	G
28	SNP	*SSC/ndhD-ccsA*	IGS	121279	G	A
29	InDel	*LSC/trnH-psbA*	IGS	167	-	AAATC
30	InDel	*LSC/rps16 intron*	gene	5417	C	-
31	InDel	*LSC/trnC-petN*	IGS	9481	T	-
32	InDel	*LSC/rpoc1-intron*	gene	16831	GGAAACTTGAGTAAGGAGTAGATC	-
33	InDel	*LSC/rpoc2-rps2*	IGS	23086	T	-
34	InDel	*LSC/psbZ-trnG*	IGS	35508	-	A
35	InDel	*LSC/trnG-trnfM*	IGS	35818	-	AGCCTTC
36	InDel	*LSC/ycf3-trnS*	IGS	43835	A	-
37	InDel	*LSC/ycf3-trnS*	IGS	44098	T	-
38	InDel	*LSC/trnL_intron*	gene	46911	-	TGTAGACATAA
39	InDel	*LSC/trnM-atpE*	IGS	52127	-	TTAAAT
40	InDel	*LSC/accD*	gene	56925	-	GTCTTG
41	InDel	*LSC/ycf4-cemA*	IGS	60146	-	AGAAAT
42	InDel	*LSC/clpP*	gene	70273	-	T
43	InDel	*LSC/petB-petD*	IGS	76172	-	TTTATTTAACATAATATAGTTGA
44	InDel	*SSC/ndhD-ccsA*	IGS	121280	ATTTTTATTC	-

* IGS = intergenic spacer region

Gene content and arrangement were identical in all three sequenced *Taraxacum* plastomes. The plastome contains 135 unique genes, including a total of 81 protein-coding genes (plus 9 duplicated in IR), 4 rRNA (all duplicated in the IR) and 38 unique tRNA genes (one in the SSC region, 23 in the LSC region and 7 duplicated in the IR region) with two copies of the *trn*F*-*GGA gene in the LSC region and four rRNA genes in the IR region (Table 2; Fig 1). Within the IRs, there are 19 genes duplicated: all four rRNA, seven tRNA and eight protein-coding genes. Only the 5' end of the *ycf*1 genes (467 bp) and 3' end of *rps*19 (67 bp) are present in the IRs, and the gene *rps*12 is trans-spliced, with the 5' exon in the LSC and the remaining two exons in the IRs (Fig 1). There are 18 different intron-containing genes (of which six are tRNA coding genes). All intronic genes contain one intron, except two (*ycf*3, *clpP*) that contain two introns. The *trn*K-UUU gene had the largest intron (2,557 bp) with another gene, *mat*K, located in it (Table 3). Sequences have been submitted to GenBank (GenBank accession number: KX499523, KX499524, KX499525), and the full raw reads from the three genotypes have been uploaded into SRA with BioSample accessions: SAMN05300515, SAMN05300516, SAMN05300517.

**Table 2:** Genes present in the *Taraxacum* plastomes.

	Category	Gene name
1	**Photosystem I**	psaA, psaB, psaC, psaI, psaJ, ycf3^a^, ycf4
2	**Photosystem II**	psbA, B, C, D, E, F, H, I, J, K, L, M, N, T, Z
3	**Cytochrome b6/f**	petA, B ^b^, D ^b^, G, L, N
4	**ATP synthase**	atpA, B, E, F^b^, H, I
5	**Rubisco**	rbcL
6	**NADH Oxidoreductase**	ndhA^b^, B^b^^,^^c^, C, D, E, F, G, H, I, J, K
7	**Large subunit ribosomal proteins**	rpl2^b^^,^^c^, 14, 16^b^, 20, 22, 23^c^, 32, 33, 36
8	**Small subunit ribosomal proteins**	rps2, 3, 4, 7^c^, 8, 11, 12^b^^,^^c^^,^^d^, 14, 15, 16^b^, 18, 19^c^
9	**RNAP**	rpoA, B, C1^b^, C2
10	**Other proteins**	accD, ccsA, cemA, clpP^a^, matK, infA
11	**Proteins of unknown function**	ycf1, ycf2^c^, ycf15^c^, ycf68^c^
12	**Ribosomal RNAs**	rRNA23^c^, 16^c^, 5^c^, 4.5^c^
13	**Transfer RNAs**	trnA(UGC)^b^^c^, trnC(GCA), trnD(GUC), trnE(UUC, trnF(GAA)^f^, trnfM(CAU), trnG(GCC), trnG(UCC)^b^, trnH(GUG), trnI(CAU)^c^, trnI(GAU)^b^^c^, trnK(UUU)^b^, trnL(CAA)^c^,trnL(UAA)^b^, trnL(UAG), trnM(CAU), trnN(GUU)^c^, trnP(UGG), trnQ(UUG), trnR(ACG)^c^, trnR(UCU), trnS(GCU), trnS(GGA), trnS(UGA), trnT(GGU), trnT(UGU), trnV(GAC)^c^,trnV(UAC)^b^,trnW(CCA), trnY(GUA)

^a^ Gene containing two introns;

^b^ Gene containing a single intron;

^c^ Two gene copies in the IRs;

^d^ Gene divided into two independent transcription units;

^f^ Duplicated gene in LSC.

**Table 3:** Intron and exon sizes in genes in the *Taraxacum* plastome.

Genes	Regions	ExonI (bp)	Intron (bp)	ExonII (bp)	IntronII (bp)	ExonIII (bp)
*atpF*	LSC	145	707	410	-	-
*ndhA*	SSC	553	1054	539	-	-
*ndhB*	IR	777	669	756	-	-
*petB*	LSC	642	769	6	-	-
*petD*	LSC	475	707	8	-	-
*rpl2*	IR	391	665	434	-	-
*rpoC1*	LSC	453	709	1638	-	-
*rps12*	LSC/IR	114	-	243	-	-
*rpl16*	LSC	408	1058	9		
*rps16*	LSC	40	860	227	-	-
*trnA(UGC)*	IR	38	814	35	-	-
*trnG(UCC)*	LSC	23	726	47	-	-
*trnI(GAU)*	IR	43	772	35	-	-
*trnK(UUU)*	LSC	37	2557	35	-	-
*trnL(UAA)*	LSC	37	440	50	-	-
*trnV(UAC)*	LSC	38	572	38	-	-
*ycf3*	LSC	124	690	230	740	153
*clpP*	LSC	71	623	291	812	229

A total of 26233 codons in S3 and O978, and 26253 codons in A978 represent the coding regions of 90 protein-coding genes. Codon usage was biased towards A and T at the third codon position. Among the codons, serine (8.8% and 8.9% of O978, A978 respectively) and methionine (1.77% and 1.80% of O978, A978 respectively) are the most and the least abundant amino acids (S2 Table).

**Fig 3:**
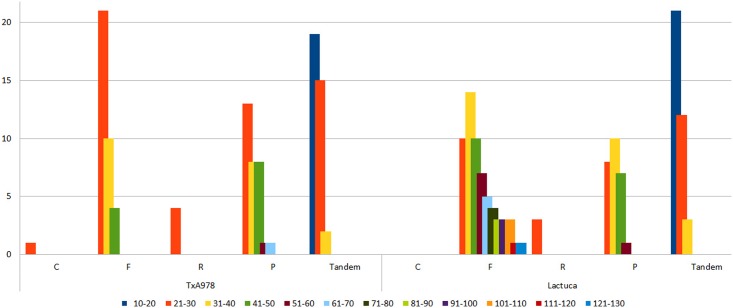
Repetitive motif abundance in *Taraxacum* (only A978 shown since the three accessions were similar) and *Lactuca* plastomes. C = Complement repeats, P = Palindromic repeats, F = Forward repeats, R = Reverse repeats.

Investigation of various types of repeats present in *Taraxacum* plastome showed the presence of five main types of repeats (complement, forward, reverse, palindromic and tandem) (Fig 3, S3 Table). The most abundant were short repeats of sequence motifs with 21–30 nucleotides, except for tandem repeats, were the most abundant were motifs with only 10–20 nucleotides. Comparison with *Lactuca* (DQ_383816) showed difference in both types of present repeats and length of repeats (Fig 3).

### Comparison of chloroplast features between *Taraxacum* and 21 accessions of Asteraceae and phylogenetic analyses

**Fig 4:**
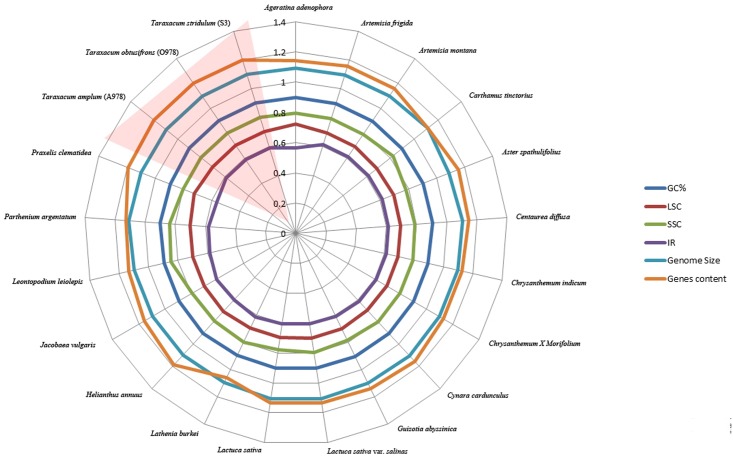
A radar-plot comparing features of the plastomes of 21 accessions of Asteraceae, showing, from inside to out, sizes of major plastome regions, GC content, genome size and number of different types of genes.

Comparison of chloroplasts between *Taraxacum* and other Asteraceae (Table 4) showed no dramatic difference in compared features (Fig 4, numerical data in S4 Table). The most prominent difference was observed in the number of genes with *Taraxacum*, together with *Helianthus annuus* (S4 Table), having the highest gene content (136 genes) from all of the compared species. Genome size, GC content and size of LSC did not vary considerably, while size of SSC was slightly bigger for two taxa (*Parthenium argentatum* and *Leontopodium leiolepis*) and of IR was lower for *Ageratina adenophora* and *Praxelis clematidea* (Fig 4).

**Table 4:** List of plastomes from GenBank used for comparison.

Sub-family	Tribe	Organism name	Ref seq.	Reference
**Asteroideae**	**Heliantheae alliance**	*Guizotia abyssinica*	NC_010601.1	[70]
		*Helianthus annuus*	NC_007977.1	[40]
		*Parthenium argentatum*	NC_013553.1	[71]
	**Anthemideae**	*Artemisia frigida*	NC_020607.1	[56]
		*Artemisia montana*	NC_025910.1	-
		*Chrysanthemum indicum*	NC_020320.1	[72]
		*Chrysanthemum X Morifolium*	NC_020092.1	[72]
	**Astereae**	*Aster spathulifolius*	NC_027434.1	[73]
	**Senecioneae**	*Jacobaea vulgaris*	NC_015543.1	[74]
	Gnaphalieae	*Leontopodium leiolepis*	NC_027835.1	-
	Eupatorieae	*Ageratina adenophora*	NC_015621.1	[64]
		*Praxelis clematidea*	NC_023833.1	[75]
	Madieae	*Lasthenia burkei*	Km360047	[61]
**Cichorideae**	**Cichorieae**	*Lactuca sativa*	NC_007578.1	[59]
		*Lactuca sativa var. salinas*	DQ_383816_	[40]
		*Taraxacum amplum (A978)*	KX499525	This paper
		*Taraxacum obtusifrons (O978)*	KX499524	This paper
		*Taraxacum stridulum (S3)*	KX499523	This paper
**Carduoideae**	Cynareae	*Centaurea diffusa*	NC_024286.1	[76]
		*Cynara cardunculus*	NC_027113.1	[77]
		*Carthamus tinctorius L.*		[57]
**Solanaceae**		*Nicotiana tabacum*	NC_001879	[8]

Based on comparison of sequences of whole plastomes, higher sequence divergence was present within non-coding regions. The most divergent coding regions between *Taraxacum* plastomes and the others 18 Asteraceae plastomes were *rpo*C1, *rpo*C2, *trnL*, *acc*D, *clp*P, *psb*B, *ndh*D, *ycf*1, *ndh*A, *rps*16 and *ndh*F (S2 Fig). Using the Maximum Likelihood method and nucleotide substitution models with minimum Bayesian information criterion (BIC) value for each tree from MEGA6 ([47]; S1 Table), 41 trees were produced. In all of them, the three Taraxacum microspecies appeared as a clade which usually (in 33 of the 41 trees) showed a well-supported sister group relationship to Lactuca. This is consistent with both genera belonging to subfamily Cichorioideae. Some DNA regions showed either a paraphyleteic (rRNA, tRNA, *trn*G-*trnf*M, *pet*A-*psb*J, *clp*P) or polyphyletic (*trn*H-*psb*A, *rpo*C2-rps2, *trn*S-*trn*C) Cichorioideae (S3 Fig), but most of these were relatively short sequences. Species in subfamily Carduoideae (belonging to the genera *Cynara*, *Centaurea* and *Carthamus*) were often sister to Cichorioideae (in 17 of the 41 trees), but there were several where other groups showed this relationship.

## Discussion

Species in the Asteraceae family have contrasting evolutionary pressures from intense selection by people in agricultural and weedy species, with presumably relaxed selection in favourable niches, and there are some invasive species with genetic bottlenecks. The species also have various breeding systems including apomixis, sporophytic self-incompatibility, cleistogamy, wind and insect pollination and there is interest in the use of more apomictic crop species. With whole plastome sequences and comparisons between families, it will be valuable to identify the nature of evolutionary processes in nuclear and cytoplasmic genomes with the different selection pressures, population structures and breeding systems. Here, we provide brief discussion of main features of *Taraxacum* plastome gained form sequencing of whole chloroplasts in three apomictic accessions.

### Chloroplast genome polymorphisms between *Taraxacum* microspecies and differentiation power of plastome sequences at low taxonomic level

The three apomictic accessions for which whole plastome sequences were generated in the present study belong to a group of common dandelions (generally called *T*. *officinale* aggregate). Sequenced individuals represent agamospermous progeny of maternal plants genotyped by nuclear markers by Majeský *et al*. [32]. This genotyping showed two defined groups (OSP and AMP) and supported the presence of nine tight genetic clusters among the nine studied apomictic accessions (for details see Majeský *et al*. [32]). The genotyping agreed with the morphologically-based division of the accessions into separate apomictic microspecies (a taxonomic rank for apomictic taxa based on morphology). Of the three apomictic microspecies sequenced in the present study, two (O978, S3) belong to the OSP group and A978 belongs to the AMP group. Despite their clear and robust nuclear differentiation, sequencing of the chloroplast *trn*L*–trn*F intergenic spacer showed they shared the cp*1a* haplotype: haplotype cp*1a* (haplotype 18a in Wittzell [35]) is the most common (derived) haplotype shared among wide spectrum of different sections (dandelion groups) in *Taraxacum* [32, 34, 35]. This suggests haplotype cp*1a* might be derived from the most recent common ancestor of many derived *Taraxacum* sections.

Van der Hulst *et al*. ([49] their Fig 3), identified three *Taraxacum* chloroplast haplotypes in more than two plants (namely C1, C2 and C4), and found these were not restricted to single clades based on nuclear marker data (AFLPs (amplified fragment length polymorphisms) and microsatellites). They were neither monophyletic nor congruent with nuclear markers, thus negating the model that matrilineal markers would delimit nuclear marker data to matrilineal groups and thus detect clonal lineages. However, this study employed population-based sampling (randomly sampled individuals within a ‘park lawn’). In such a habitat many different morphological clones (microspecies) coexist (see e.g. [50, 51]) with different origin. In the case of apomicts, like *Taraxacum*, nuclear markers are able to delimit clonal lineages [32, 52] and the extent of a clonal lineage can be supported by matrilineal markers, although not unambiguously, (e.g. see Majeský *et al*. [34]). However, the markers used only consider a small fraction of the whole chloroplast and inevitably cannot discover all differences within particular chloroplast lineages. Whole plastome sequencing of well-defined samples measured all genetic variability among the three apomictic dandelions. The plastome sequences were identical in the two apomictic accessions O978 and S3, belonging to same morphological group OSP, and differed by 27bp in length, 28 SNPs and 16 indels from A978, belonging to different AMP group (Table 1).

What do these results show about the relationship between the apomictic microspecies where we sequenced the plastome, following the work of Majeský *et al*.[32] Plastomes are evolving at a different, slower rate, compared to nuclear markers, as noted by Wolfe [53]. While nuclear markers showed genetic boundaries between the O and S sub-groups, whole chloroplast sequences did not. This may point to the young evolutionary age of the two microspecies (*T*. *obtusifrons* and *T*. *stridulum*): they have not accumulated any chloroplast mutations between each other and their most recent common ancestor. Morphologically, they are well-defined as separate morphological units [32] with a low number of observed genotypes within investigated individuals from the O and S microspecies: two (*T*. *obtusifrons*) and four (*T*. *stridulum*) multilocus genotypes were detected by six nuclear SSRs (simple sequence repeats) among 21 and 23 genotyped individuals, while AFLPs showed only one AFLP-phenotype among 10 fingerprinted individuals of both microspecies. Apomictic reproduction cuts off a lineage from genetic recombination so an asexual lineage is expected to rapidly diverge as a result of accumulation of mutations and transposon activity that become the major generators of diversity and driver for genome evolution [54, 55].

### Comparison of *Taraxacum* plastome with other genera

**Fig 5:**
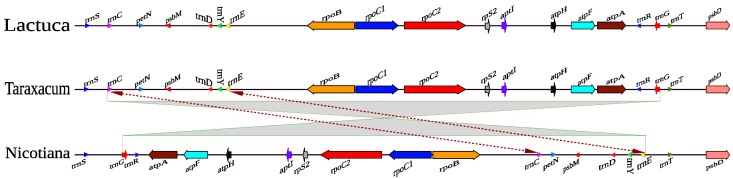
Comparative plastome maps. Endpoints of the large 22 kb inversion present in most Asteraceae and of a small inversion (3.3 kb in other Asteraceae).

Sequence comparison of the plastome of *Taraxacum* with the reference *Nicotiana tabacum* [8] revealed hemi-nested inversions in the LSC region (Inv1: 21,737 bp in S3/O978, and 21,711 bp in A978; inv2 of 2,543 bp in S3/O978 and 2,542 bp in A978; Figs 1 and 2). The nested inversion ended just upstream of the *trn*E*-*UUC gene with the large inversion. The other end-point of the inversion is located between the *trn*C*-*GCA and *rpo*B genes (Fig 5). The inversion in the LSC (Inv1 and Inv2; [23, 40]) is conserved across all 21 Asteraceae chloroplast sequences. Liu *et al*. [56] suggested that the LSC inversion region has undergone inversion followed by reinversion in Asteraceae, and that this could be a particularly active region for sequence rearrangements in the plastome: the existence of within-species variation in the presence of this major inversion supports the hypothesis that this region is a hotspot for inversion events (Fig 5).

**Fig 6:**
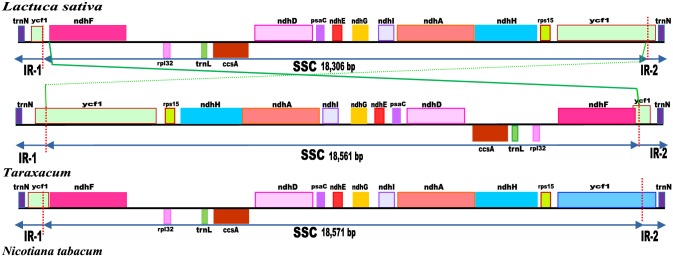
Comparative plastome maps. **Gene order and inversion of the SSC region**. Gene sequences were annotated and indicated along the black lines. Genes above the black lines indicate their transcription in reverse direction and genes below the black lines represent their transcription in forward direction.

**Fig 7:**
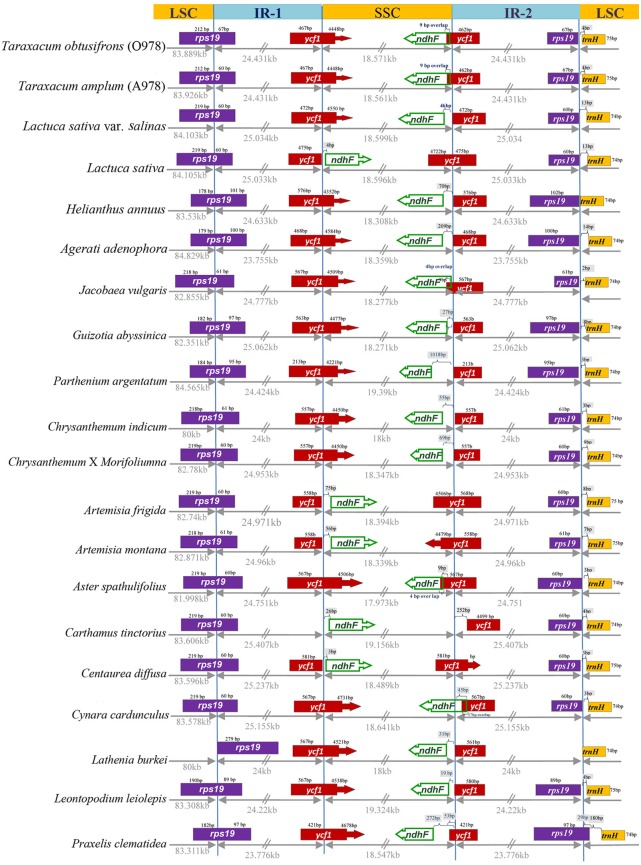
Comparative plastome maps. **Border position of LSC, IR and SSC region among the 20 Asteraceae plastomes.** Genes are indicated by coloured boxes.

Another large inversion between *N*. *tabacum* and *Taraxacum* (Figs 2 and 6) is present between base pair positions 108321 in S3, O978 (108,358 in A978) and 126891 in S3, O978 (126,919 in A978); it is flanked by inverted repeats and encompasses the entire SSC region (18,571 bp in S3, 18,561 bp in A978) (Figs 2, 6 and 7). The SSC inversion from *ndh*F to *rps15* is present in all of the Asteraceae lineages involved in this study except *Artemisia frigida* (NC_020607) [56], *Artemisia montana* (NC_025910), *Carthamus tinctorius* (KP404628) [57], *Centaurea diffusa* (NC_024286) [76] and one reported *Lactuca sativa* (NC_007578) [59].

Comparison of features of the plastomes of 21 accessions of Asteraceae, showed overall similarity of chloroplasts across wider spectrum of different evolutionary lineages. There were even no dramatic differences among representatives of the three main subfamilies (Carduoidae, Cichorioidae, Asteroidae), what may stress overall high stability of chloroplast features at lower taxonomic level (S4 Table, Fig 4). The most remarkable difference was seen in the number of total tRNA and coding genes (S4 Table), with *Lasthenia burkei* being taxon with the lowest number of genes (119—Total Gene N°/79—N° Coding Genes/20—N° tRNA) comparing with the three *Taraxacum* (136 –Total Gene N°/90—N° Coding Genes/38—N° tRNA). Holmquist [60] considered that recombinogenic domains of chromosomes may be GC rich. Fig 1 shows that the GC content was lower in the SSC region flanked by IR1 and IR2, and higher in 7kb (of the 24kb) of the IR regions 1kb away from the SSC border, with an evident spike from low GC at end of both IRs; both ends of Inv1 had a low GC content. Thus, as found by Walker *et al*. [61], high GC content was not associated with inversion breakpoints in the plastome.

The number of direct (forward), reverse, palindromic and tandemly repeated sequence motifs of various length classes in *Taraxacum*, compared with *Lactuca* (DQ_383816), can be seen in Fig 3 (see also S3 Table). The notable difference was the increased frequency of direct repeats more than 50bp long in *Lactuca*, where there were 27 compared to none in *Taraxacum* (37 compared to 4 repeats >40bp long). Liu *et al*. [56] commented on variation in number and variety of repeats in the Asteraceae plastomes. Repeats have a role in plastome organization, but like Liu *et al*. [56], we found no correlation between large repeats and rearrangement endpoints. Our comparative repeat analysis showed considerable variation between even *Taraxacum* and *Lactuca*, with many more direct repeats of 40bp or more in *Lactuca* (Fig 3; 1% larger plastome than *Taraxacum*). Relationships of repeats and mutation have been considered in chloroplast genomes [58], although in the related *Taraxacum* plastomes, SNPs and non-repeat indels showed little relationship with repeats.

### Phylogenetic utility of chloroplast regions

Polymorphisms between the two *Taraxacum* plastomes and between *Taraxacum* and other Asteraceae included many chloroplast regions widely used for phylogenetic analysis. The presence of two *trn*F-GAA genes duplicated in the LSC is unusual and would make this region difficult to use for phylogeny and diversity studies (S4 Fig). Duplication of *trn*F-GAA gene was encountered already by Wittzell [35], who, based on sequence variation of *trn*L-*trn*F region in number of different *Taraxacum* taxa, provide support for the informal division of dandelions on evolutionarily old and evolutionary younger/derived taxa. The presence of duplicated *trn*F gene is not specific only for *Taraxacum*, but is present also in other compared species of Asteraceae: namely in *Carthamus tinctorius*, *Guizotia abyssinica*, *Ageratina adenophora*, *Praxelis clematidea* and *Lasthenia burkei* (S4 Fig). Thus, duplication of the *trn*F-GAA gene probably occurred several (at least three or four) times independently in the three main Asteraceae subfamilies: Asteroideae, Cichorideae, and Carduoideae.

All three investigated apomictic *Taraxacum* microspecies represented separate clade sister to *Lactuca* in all phylogenetic analyses (S3 Fig). This was expected because *Taraxacum* and *Lactuca* belong to the same evolutionary lineage—Cichorioideae—within the Asteraceae family (no other species of Cichorioideae was included). This is also in accordance with the current knowledge of the relationships within the subfamily [62]. Although the close relationships of both genera, *Taraxacum* represent a distinct evolutionary lineage (Crepidinae) than *Lactuca* (Lactucinae) [62] which according to Tremetsberger *et al*. [63] have diverged during the Miocene, at least 16.2 MYA. Because of low level of sequence divergence between the investigated *Taraxacum* accessions and because these microspecies represent only a scant part of species known in the genus, it is not possible to draw some conclusions about their evolutionary relationships. In part of the phylograms accession A978 appeared to be basal to O978/S3, but other phylograms do not support this and the relations between the plastomes appeared as unresolved. Definitely, whole plastome sequences provide far more discrimination power than individual markers, for phylogeny reconstruction. For deeper insight into the evolution of the *Taraxacum* genus, it will require wider sampling of more distinct taxa. Kirschner *et al*. [36] used a parsimony analysis of morphological and chloroplast data (two intergenic spacers *psb*A-*trn*H + *trn*L-*trn*F) in *Taraxacum* to show an overall lack of congruence. They suggested the conflict was a consequence of reticulation affecting morphology (and presumably nuclear markers), a process unlikely for the chloroplast genomes. Intergenic spacer *psb*A-*trn*H belonged among the most divergent plastome regions (in the sense of sequence divergence between the two distinct plastomes A978 versus O978/S3) in our analyses (presence of one SNP and 5bp InDel; Table 1), but as noted above, no sequence variation was observed among the three investigated accessions for the *trn*L*-trn*F intergenic spacer.

Both the more conserved coding regions and variable non-coding regions of the chloroplast genome have proved useful for phylogenetic studies [61, 64], with faster rates of evolution in noncoding regions; however the data here show care is needed in interpretation based on single regions as might be amplified by ‘barcode’ markers. Maybe some incongruences arise where mutations are reiterated (similarities are not identical by descent), although rare male chloroplast transmission (e.g. [65, 66]) and recombination events cannot be ruled out.

It is important to select marker sequences which have a rate of evolution that is appropriate to the evolutionary distance of the accessions under analysis and the questions being addressed [67]. Walker *et al*. [61] have pointed out that rates of molecular evolution vary over the plastome, particularly in noncoding regions. Here, two of the plastomes, from accessions which are in well-defined clades based on morphology and nuclear DNA markers, were identical: without the full plastome sequence, there would always have been questions about whether the plastome markers we happened to use were appropriate. It was also evident that the most frequently used chloroplast markers (including *trn*L-*trn*F, and *mat*K) showed few polymorphisms between O/S and A *Taraxacum* and to position *Taraxacum* with respect to other species.

*Taraxacum* microspecies, and of the species in the Cichorieae. This would enable comparisons of evolutionary rates of sexual and apomictic species, and between nuclear and plastome sequences. Tremetsberger *et al*. [63] used fossil-calibration based on pollen and a nuclear sequence to estimate divergence between species in the group, but the prehistoric and fossil record for the majority of the Asteraceae, including *Taraxacum*, is poor [63, 68, 69].

## Conclusion

We expect whole genome sequencing [61] to be used increasingly for taxonomy and systematics, within-species biodiversity, population, phylogenetic and evolutionary projects. With the total cellular DNA used here, without enrichment for chloroplast sequences, 3.5 to 4% of reads mapped to the chloroplast (400 unreplicated plastomes per 1C (unreplicated haploid) nuclear genome), allowing robust assembly including the duplications and inversions. Even with automation, PCR amplification and sequencing of multiple regions of chloroplasts and nuclear plastomes is time-consuming and requires optimization, while whole plastome sequencing only requires DNA extraction and a service provider. Analysis and interpretation of whole-genome-sequencing results is, however not yet optimized nor routine.

In the current study, we sequence full chloroplast of three well characterized apomictic *Taraxacum* microspecies. We provide the full annotated plastome sequences for the genus, which can be used in diverse spectrum of further comparative analyses and provide reference plastome for primer design in taxonomic and phylogenetic studies of the genus. We also showed the low sequence divergence between the investigated apomictic taxa, what point to their recent origin (probably post-Pleistocenic). The sequenced plastome (A978) may represent the most common recent chloroplast type involved in origin of many evolutionarily younger *Taraxacum* taxa.

## Supporting information

S1 Fig Alignment of *trn*L-UAA sequence from 19 Asteraceae species including the two *Taraxacum* (A978 and O978) species sequenced in the present study. Arrowhead indicates a 22bp insertion in A978 with respect to O978 and other species.
(TIF)

S2 Fig Comparison of plastome sequences of 18 Asteraceae accessions, two *Taraxacum* plastomes generated in this study and 16 previously reported plastomes using mVISTA program. The Y-scale represents the percent of identity ranging from 50 to 100%. Arows above the graphs indicate the direction of transcription.
(PDF)

S3 Fig Phylogenetic trees derived from maximum likelihood analysis of alignments of DNA sequences of 21 different Asteraceae species of a total of whole plastome and 40 different chloroplast regions indicated below the trees. Numbers above node are bootstrap support values.
(PDF)

S4 Fig Alignment of *trn*F-GAA sequence of investigated Asteraceae. (TIF)

S1 Table Maximum Likelihood fits of 24 different nucleotide substitution models for 22 accessions using the whole chloroplast genome plus 40 genic and inter-genic regions. Evolutionary analyses were conducted in MEGA6 [47]. Models with the lowest BIC scores (Bayesian Information Criterion) are considered to describe the substitution pattern the best, and were used for the trees in S3 Fig. As noted in MEGA6, “non-uniformity of evolutionary rates among sites may be modelled by using a discrete Gamma distribution (+G) with 5 rate categories and by assuming that a certain fraction of sites are evolutionarily invariable (+I). Whenever applicable, estimates of gamma shape parameter and/or the estimated fraction of invariant sites are shown. For estimating ML values, a tree topology was automatically computed. All positions with less than 95% site coverage were eliminated. That is, fewer than 5% alignment gaps, missing data, and ambiguous bases were allowed at any position.” There were a total of 136267 positions in the whole genome dataset, and the number of positions in the separate alignments for each region is shown (total number of positions in the dataset). Abbreviations: GTR: General Time Reversible; HKY: Hasegawa-Kishino-Yano; TN93: Tamura-Nei; T92: Tamura 3-parameter; K2: Kimura 2-parameter; JC: Jukes-Cantor.
(XLSX)

S2 Table Codon usage and codon-anticodon recognition pattern of the 21 Asteraceae plastomes calculated by http://www.bioinformatics.org/sms2/codon_usage.html. Absolute numbers and values recalculated as per mille (1/1000) and proportion are shown with a heat map gives relative usage of each codon.
(XLSX)

S3 Table Repetitive motif abundance in *Taraxacum* and *Lactuca* plastomes computed by Reputer and Tandem Repeat Finder. (XLSX)

S4 Table Characteristics of plastomes of 21 different accessions of 16 Asteraceae genera. (XLSX)
