# Supplementary figures and images for: Complete chloroplast genomes from apomictic Taraxacum (Asteraceae): Identity and variation between three microspecies
Source: PLoS One. 2017 Feb 9;12(2):e0168008. doi: 10.1371/journal.pone.0168008 (PMC5300115; doi:10.1371/journal.pone.0168008)

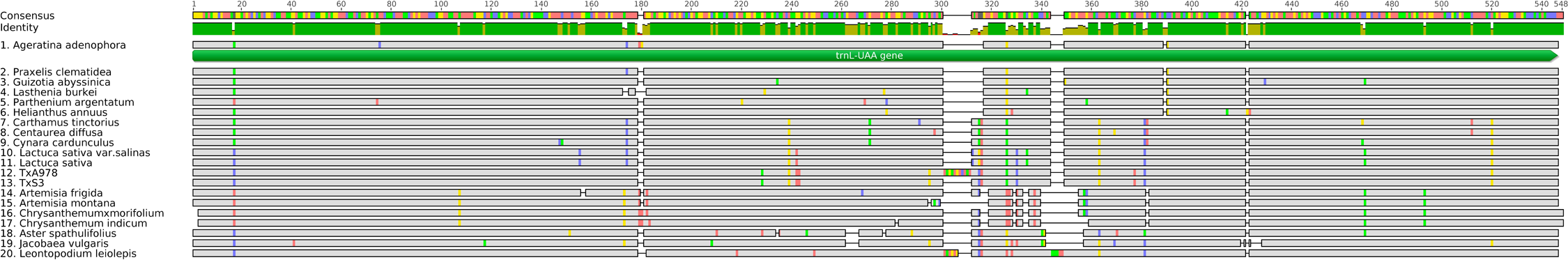

Supplement: S1 Fig — Arrowhead indicates a 22bp insertion in A978 with respect to O978 and other species. (TIF) [file pone.0168008.s001.tif]

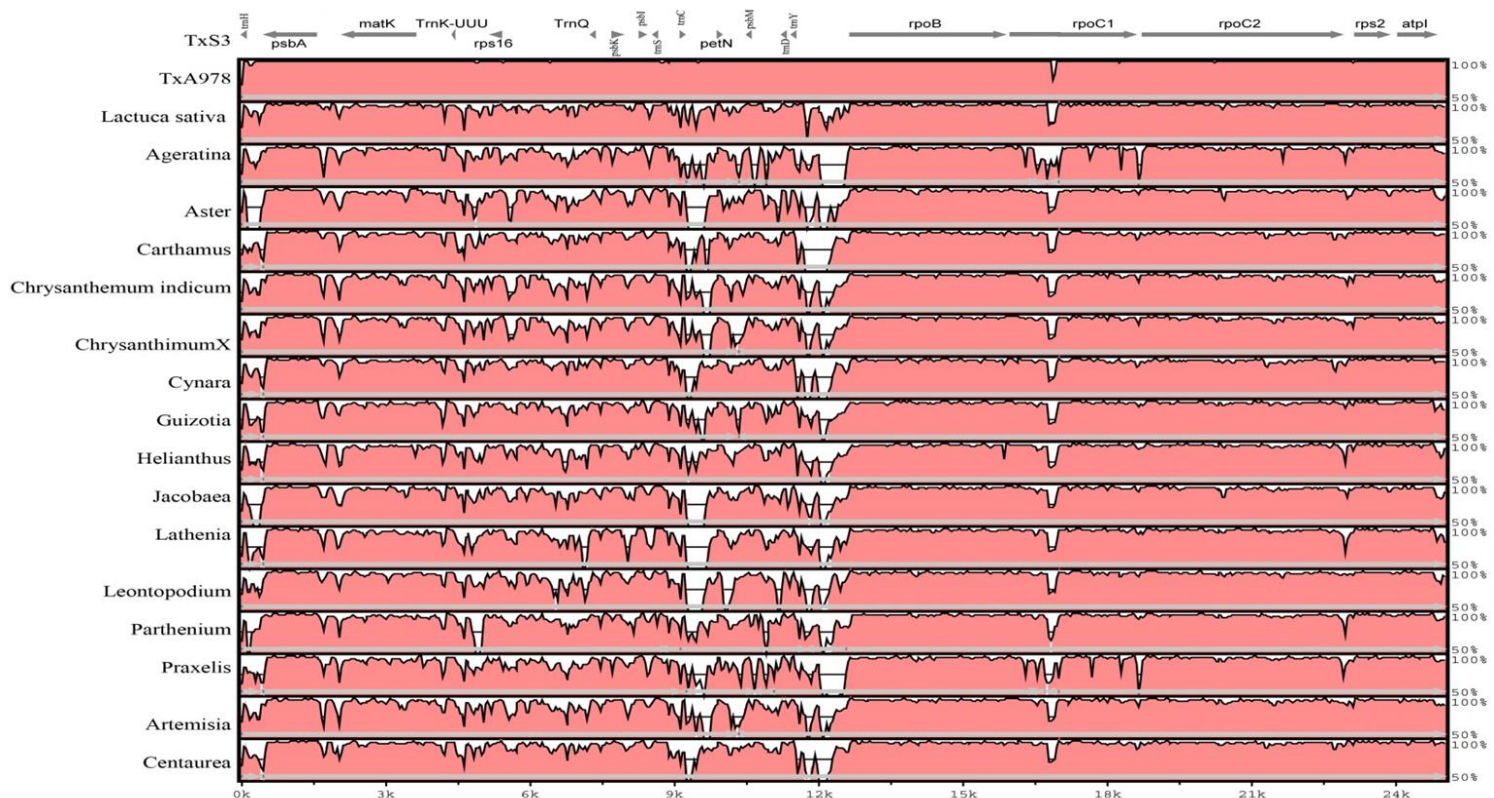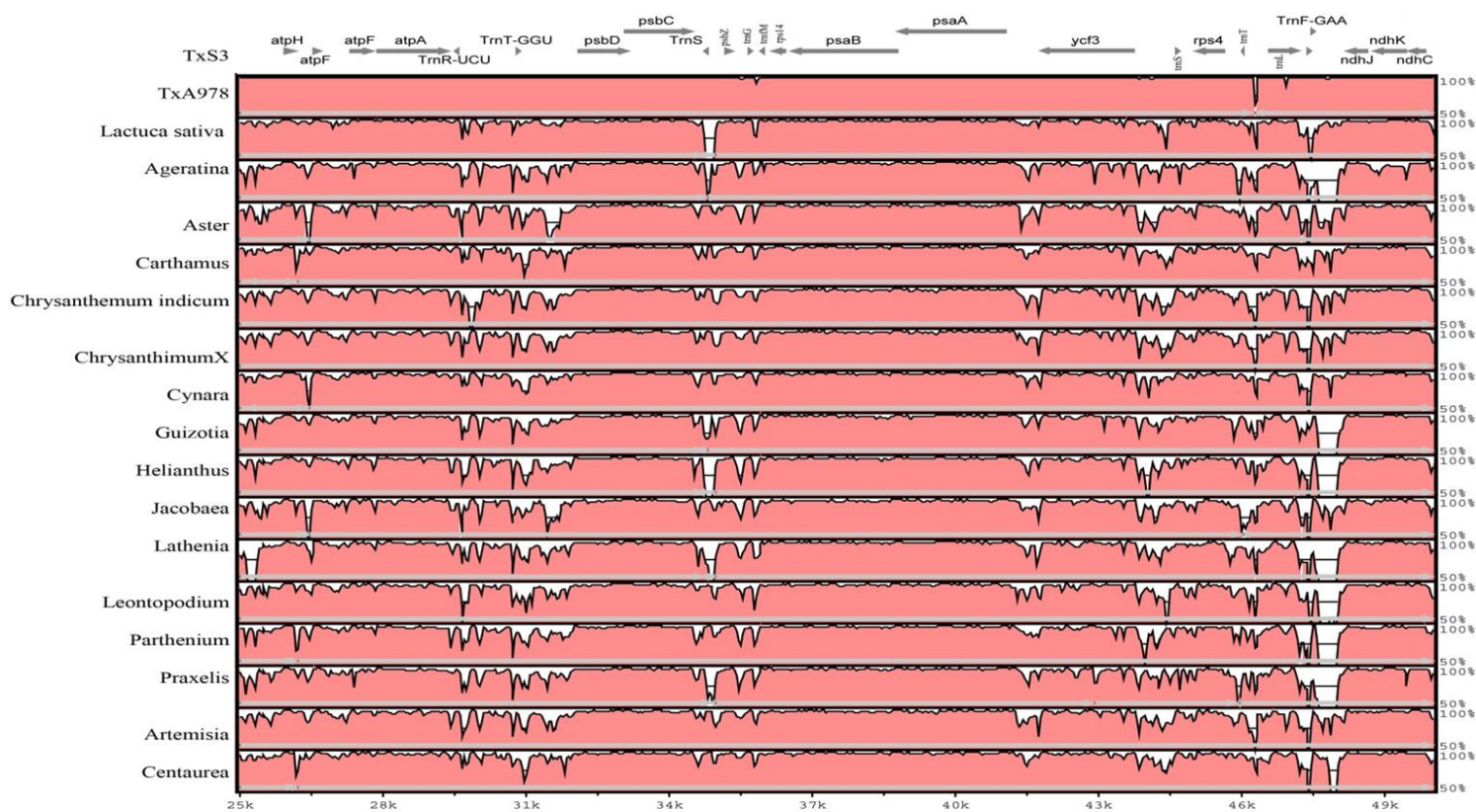

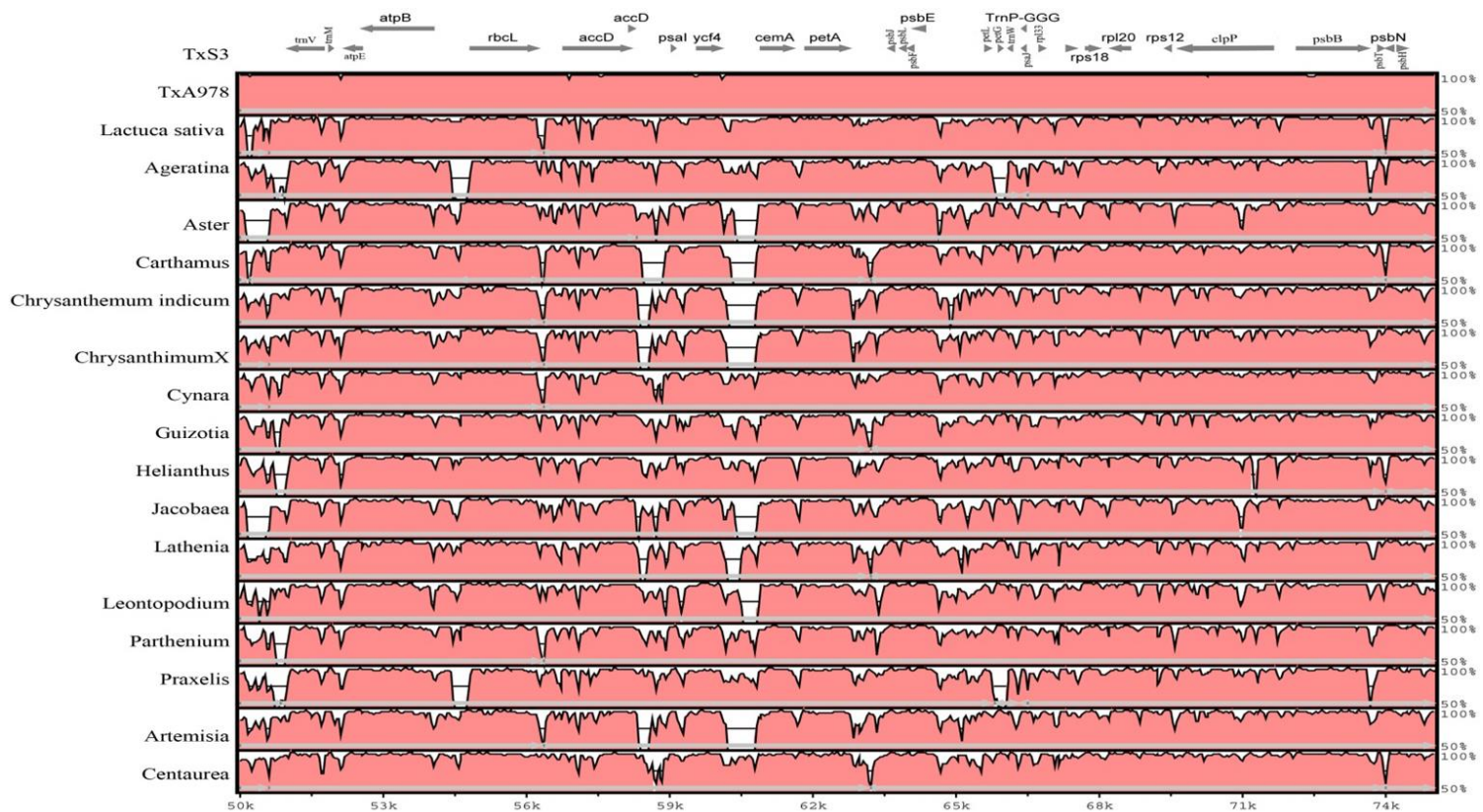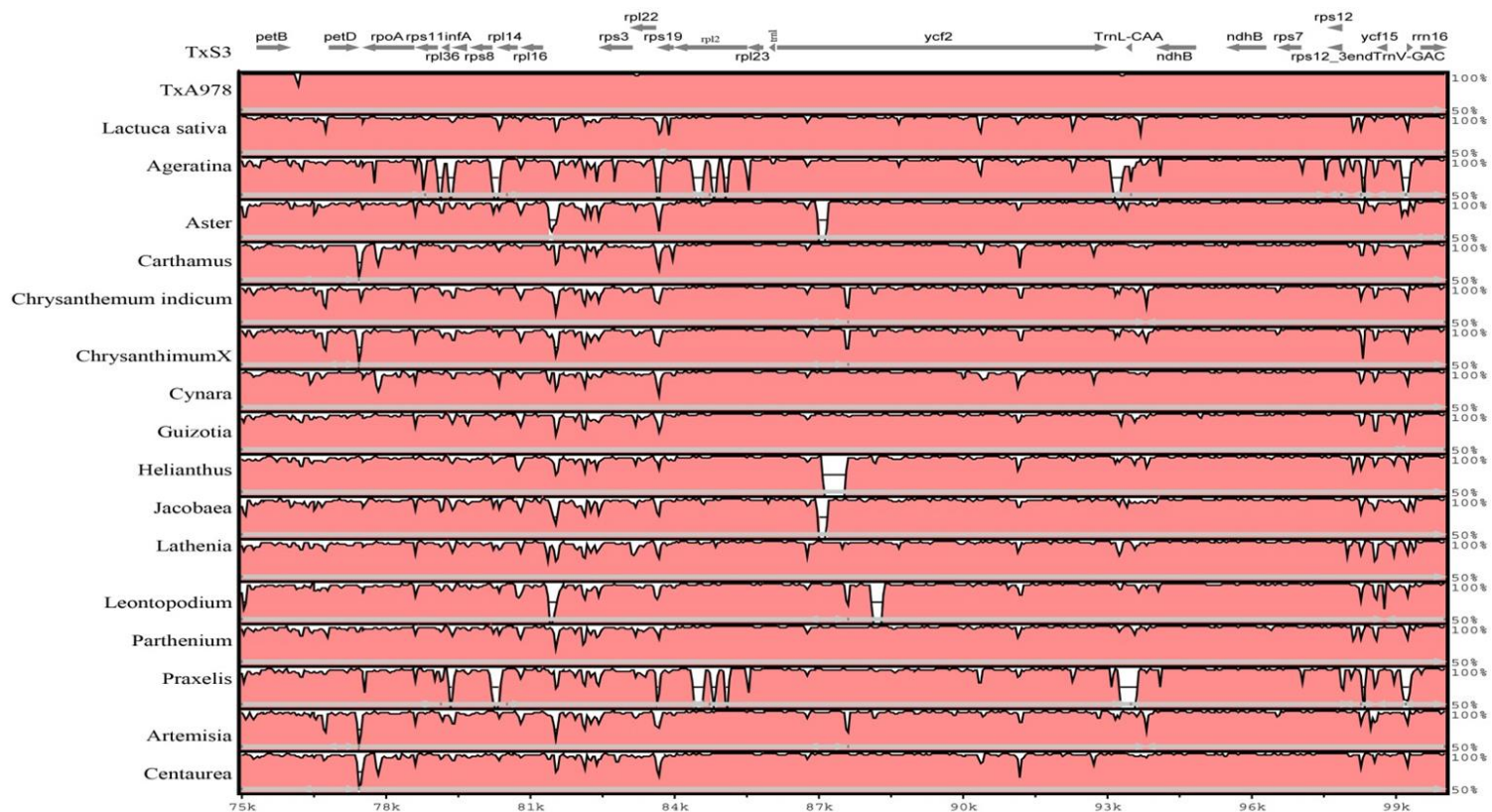

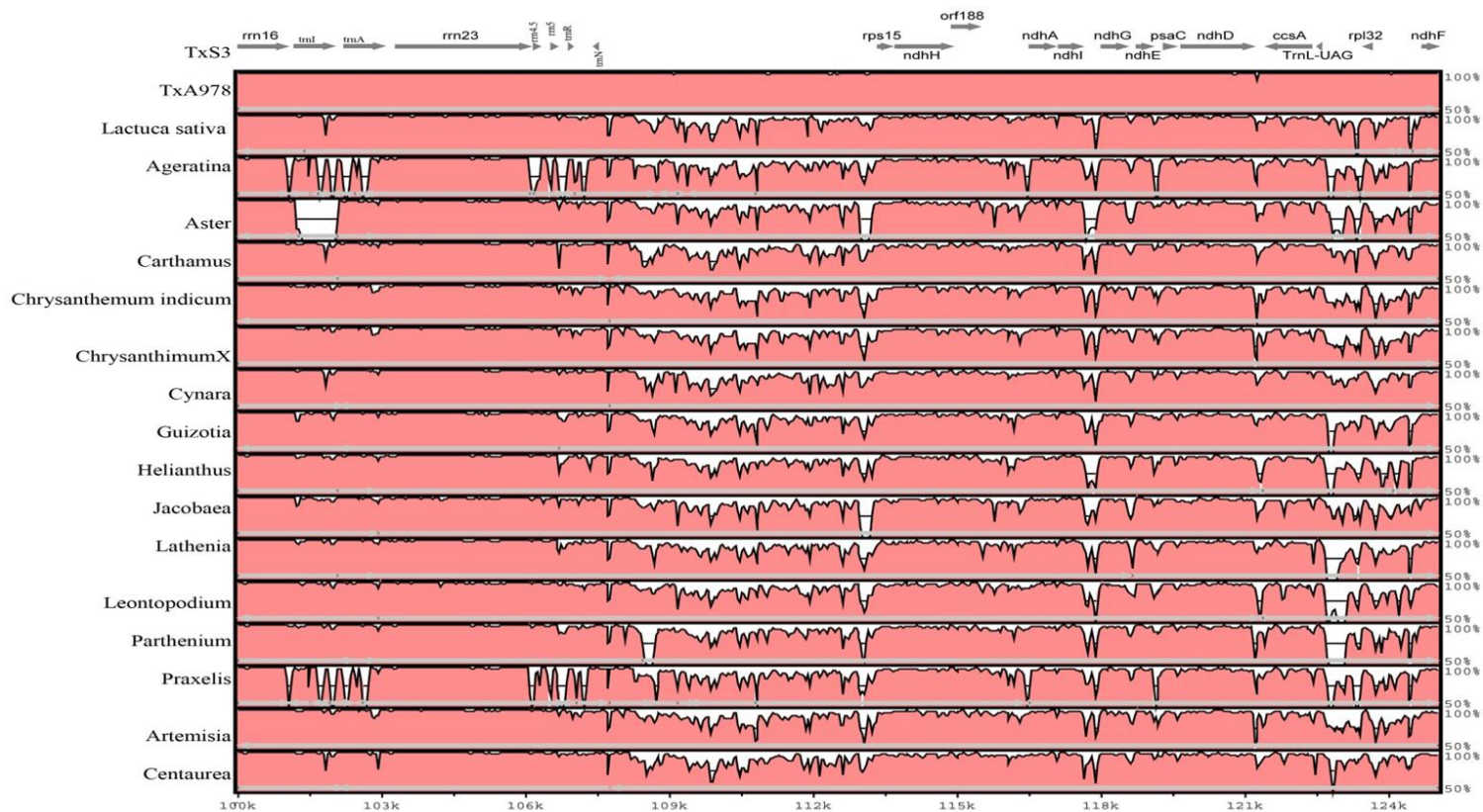

Supplement: S2 Fig — The Y-scale represents the percent of identity ranging from 50 to 100%. Arows above the graphs indicate the direction of transcription. (PDF) [file pone.0168008.s002.pdf]

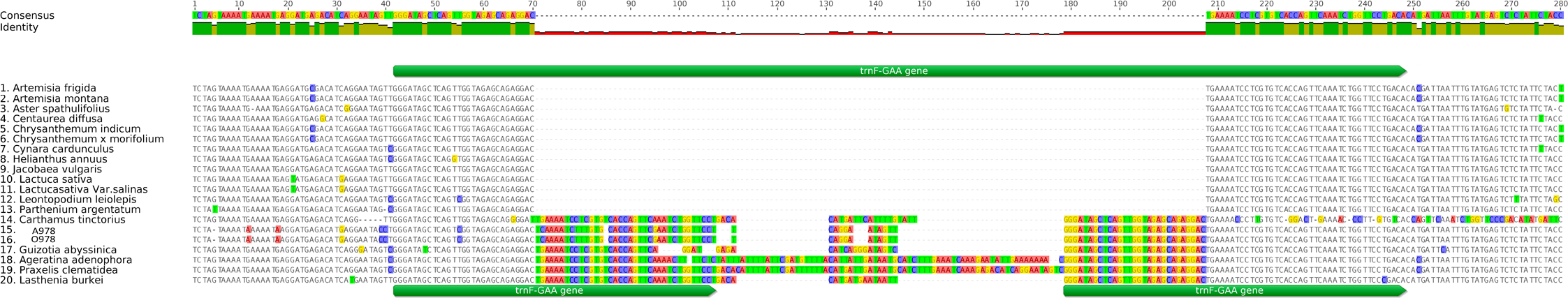

Supplement: S4 Fig — (TIF) [file pone.0168008.s004.tif]
