# Supplementary material for: Complete chloroplast genomes from apomictic Taraxacum (Asteraceae): Identity and variation between three microspecies
Source: PLoS One. 2017 Feb 9;12(2):e0168008. doi: 10.1371/journal.pone.0168008 (PMC5300115; doi:10.1371/journal.pone.0168008)
Supplement: S3 Fig — Numbers above node are bootstrap support values. (PDF) [file pone.0168008.s003.pdf]

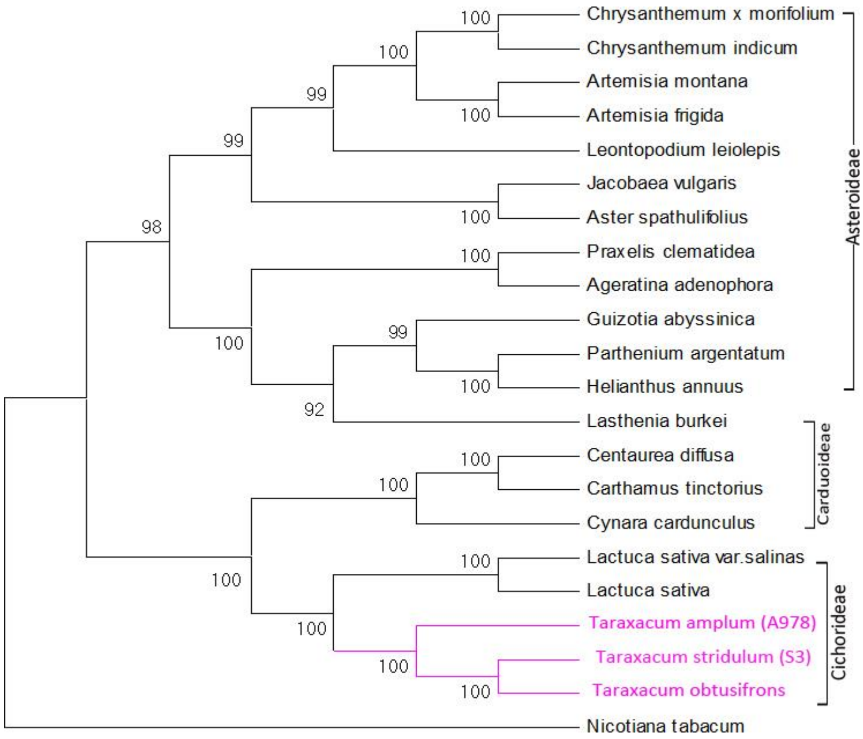

Whole chloroplast genome

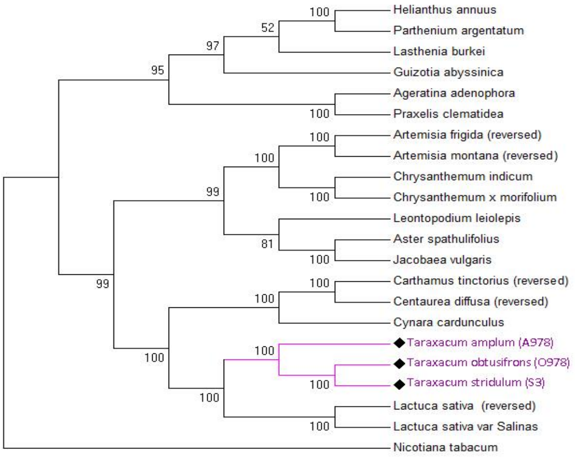

SSC region

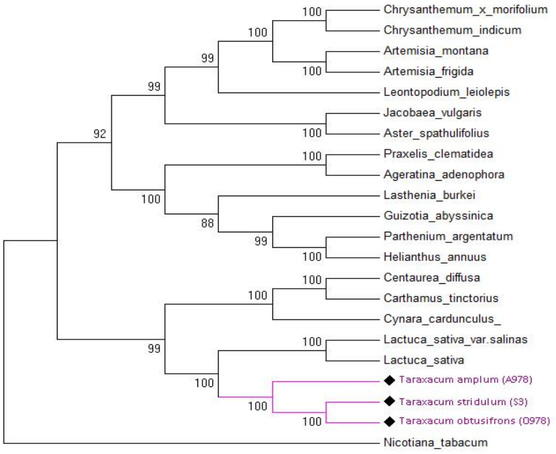

LSC region

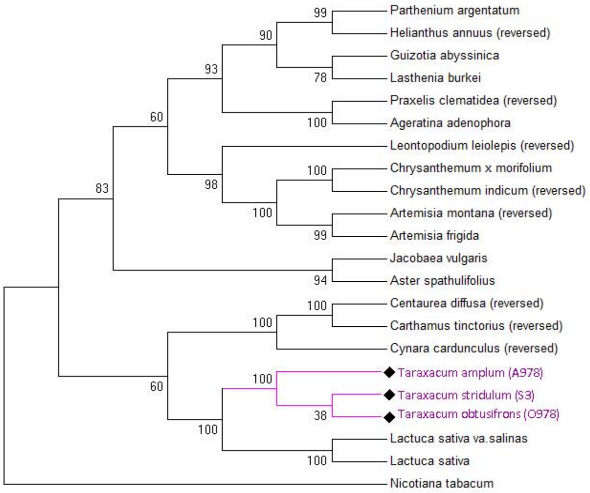

IR region

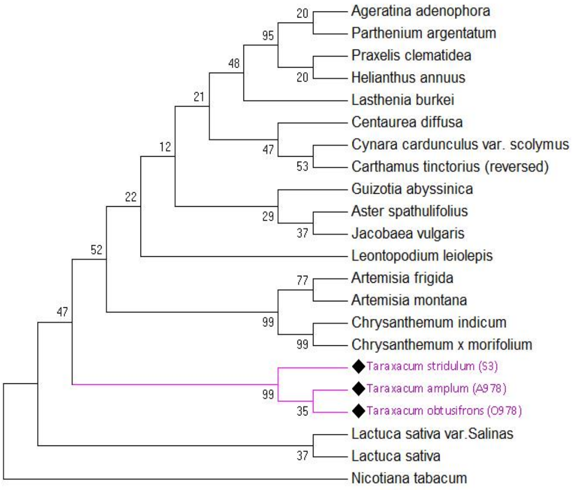

rRNA regions

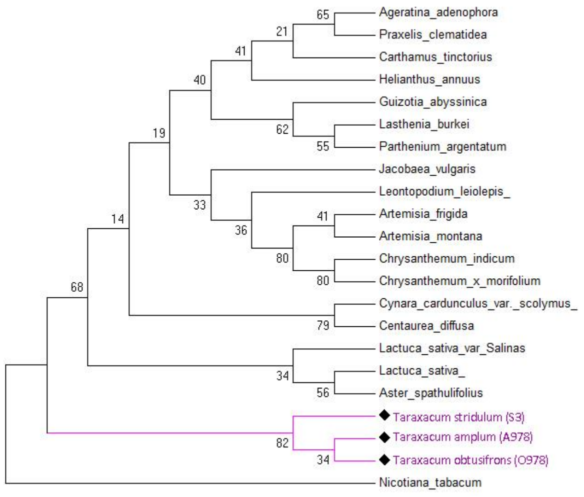

tRNA

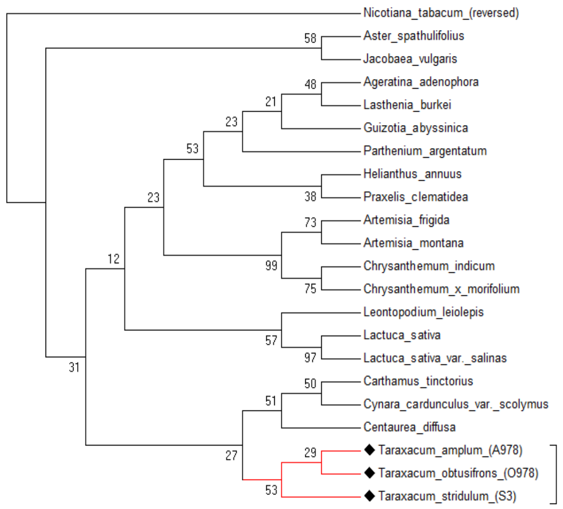

*rpo*C2-*rps*2

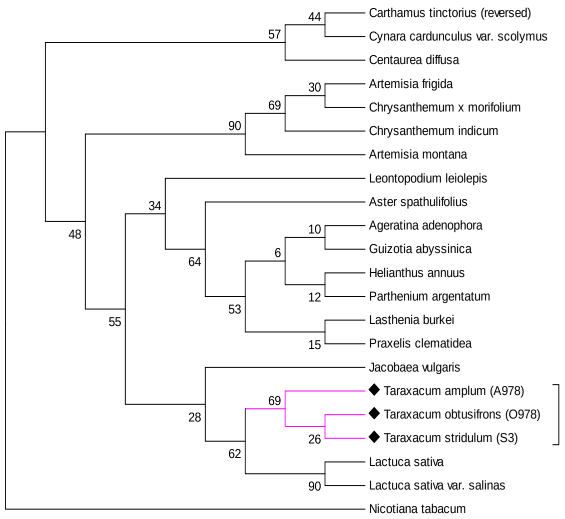

*trn*N-*ycf*1

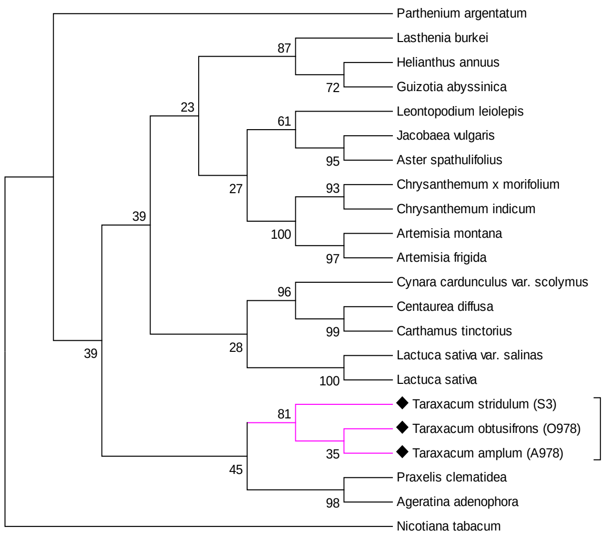

*trn*S-*trn*C

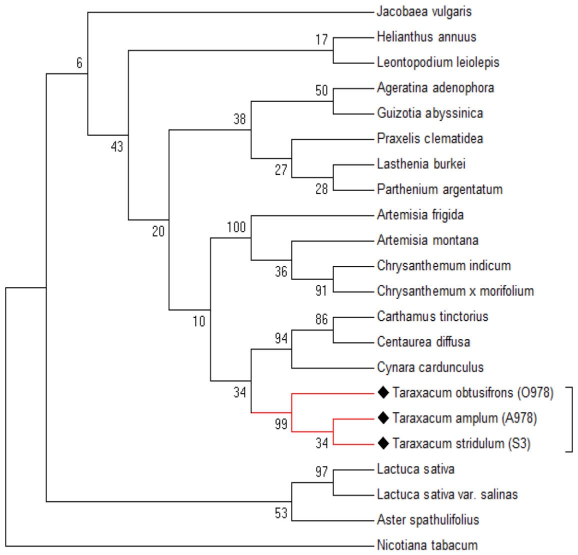

*trn*H-*psb*A

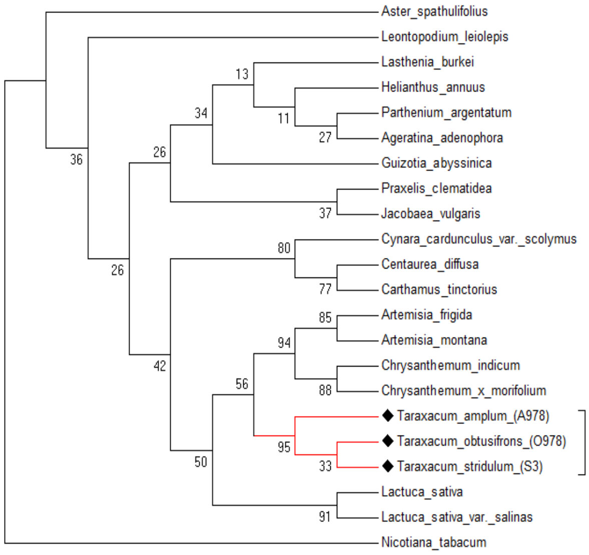

*trn*G-*trnf*M

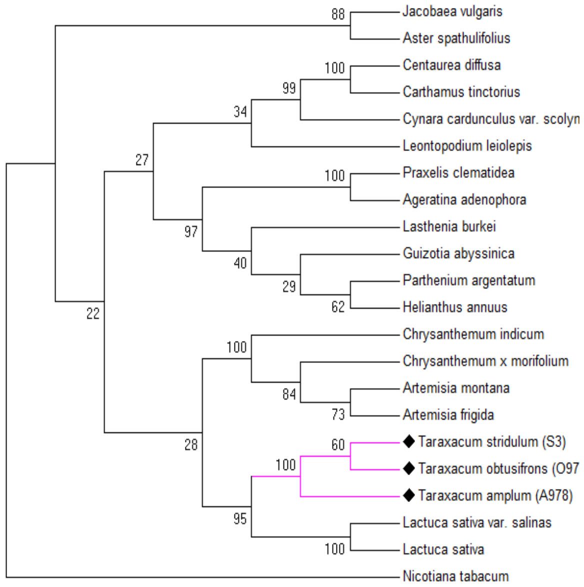

*ycf*3-*trn*S

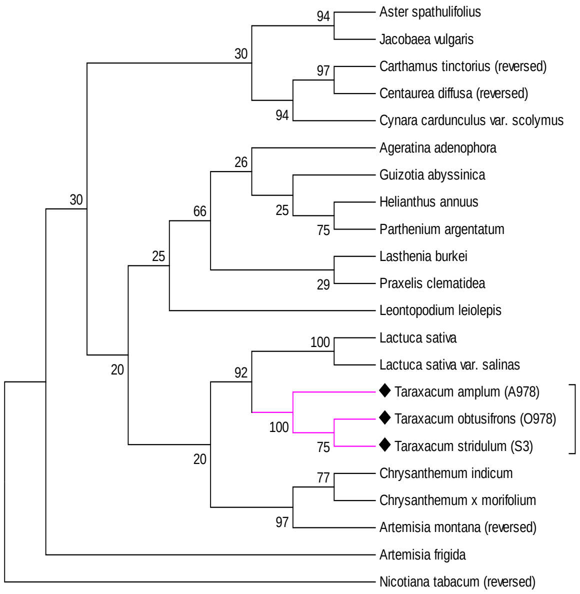

*ycf*1-*rps*15

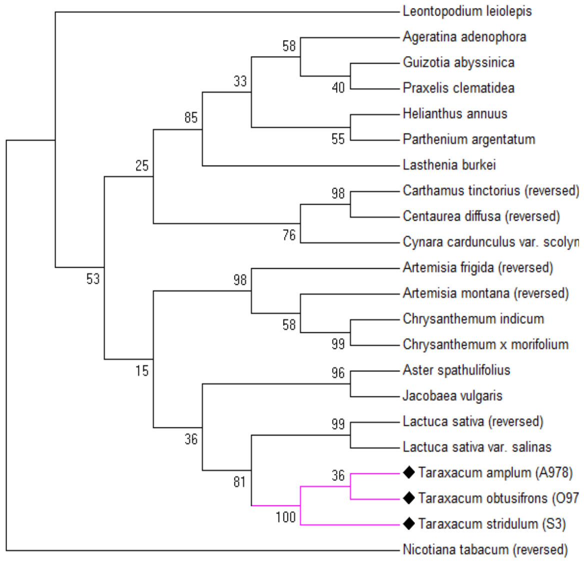

*ndh*I-*ndh*G

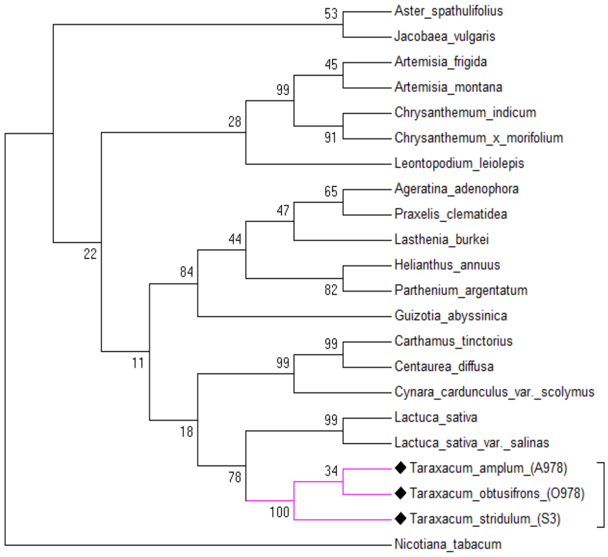

*trn*T-*trn*L

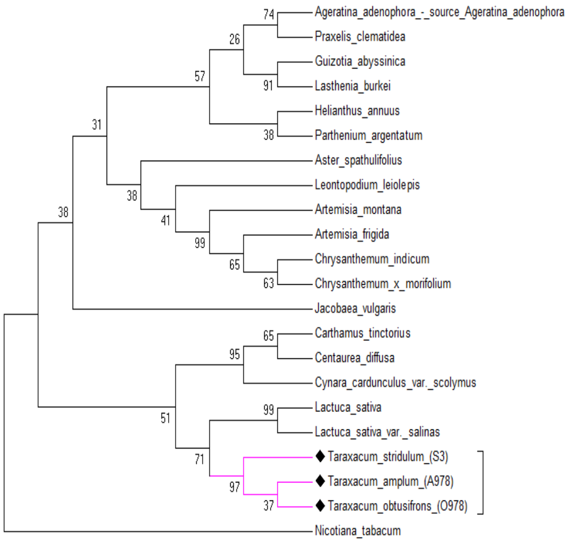

*psb*I-*trn*S

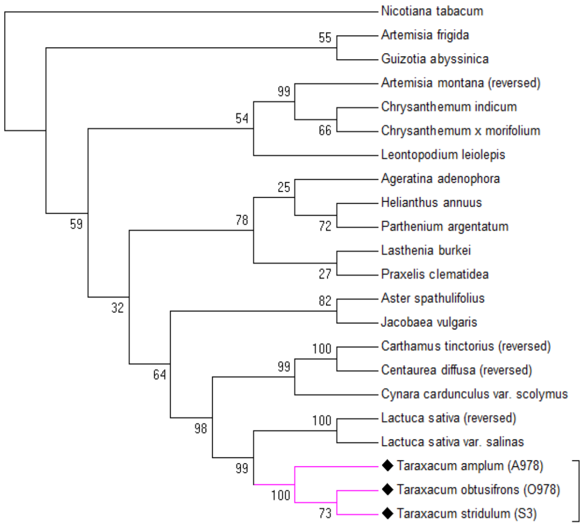

*rpl*32-*ndh*F

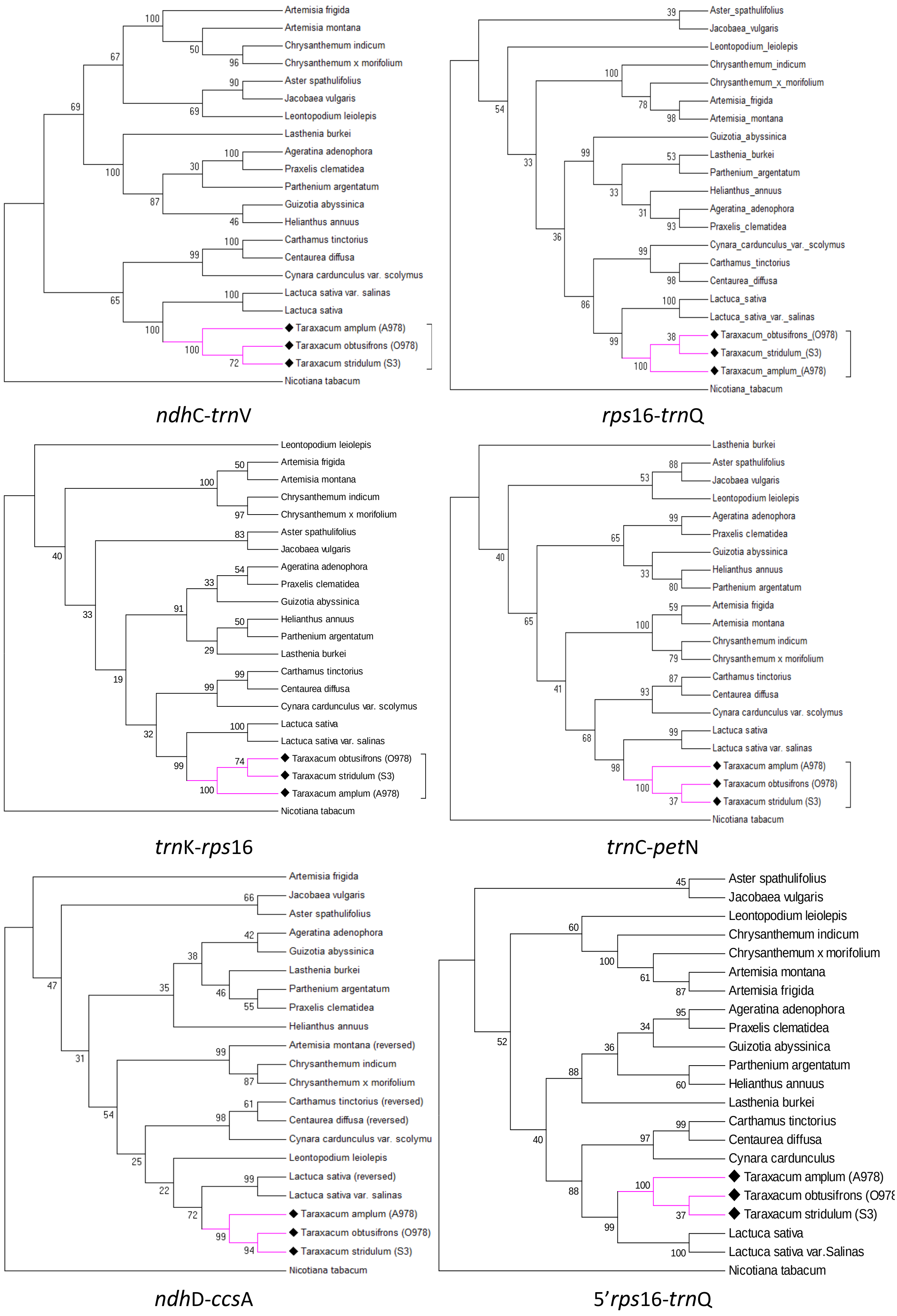

Artemisia frigida
Artemisia montana
Chrysanthemum indicum
Chrysanthemum x morifolium
Aster spathulifolius
Jacobaea vulgaris
Leontopodium leiolepis
Lasthenia burkei
Ageratina adenophora
Praxelis clematidea
Parthenium argentatum
Guizotia abyssinica
Helianthus annuus
Carthamus tinctorius
Centaurea diffusa
Cynara cardunculus var. scolymus
Lactuca sativa var. salinas
Lactuca sativa
Taraxacum amplum (A978)
Taraxacum obtusifrons (O978)
Taraxacum stridulum (S3)
Nicotiana tabacum
ndhC-trnV
Aster_spathulifolius
Jacobaea_vulgaris
Leontopodium_leiolepis
Chrysanthemum_indicum
Chrysanthemum_x_morifolium
Artemisia_frigida
Artemisia_montana
Guizotia_abyssinica
Lasthenia_burkei
Parthenium_argentatum
Helianthus_annuus
Ageratina_adenophora
Praxelis_clematidea
Cynara_cardunculus_var._scolymus
Carthamus_tinctorius
Centaurea_diffusa
Lactuca_sativa
Lactuca_sativa_var._salinas
Taraxacum_obtusifrons_(O978)
Taraxacum_stridulum_(S3)
Taraxacum_amplum_(A978)
Nicotiana_tabacum
rps16-trnQ
Leontopodium leiolepis
Artemisia frigida
Artemisia montana
Chrysanthemum indicum
Chrysanthemum x morifolium
Aster spathulifolius
Jacobaea vulgaris
Ageratina adenophora
Praxelis clematidea
Guizotia abyssinica
Helianthus annuus
Parthenium argentatum
Lasthenia burkei
Carthamus tinctorius
Centaurea diffusa
Cynara cardunculus var. scolymus
Lactuca sativa
Lactuca sativa var. salinas
Taraxacum obtusifrons (O978)
Taraxacum stridulum (S3)
Taraxacum amplum (A978)
Nicotiana tabacum
trnK-rps16
Lasthenia burkei
Aster spathulifolius
Jacobaea vulgaris
Leontopodium leiolepis
Ageratina adenophora
Praxelis clematidea
Guizotia abyssinica
Helianthus annuus
Parthenium argentatum
Artemisia frigida
Artemisia montana
Chrysanthemum indicum
Chrysanthemum x morifolium
Carthamus tinctorius
Centaurea diffusa
Cynara cardunculus var. scolymus
Lactuca sativa
Lactuca sativa var. salinas
Taraxacum amplum (A978)
Taraxacum obtusifrons (O978)
Taraxacum stridulum (S3)
Nicotiana tabacum
trnC-petN
Artemisia frigida
Jacobaea vulgaris
Aster spathulifolius
Ageratina adenophora
Guizotia abyssinica
Lasthenia burkei
Parthenium argentatum
Praxelis clematidea
Helianthus annuus
Artemisia montana (reversed)
Chrysanthemum indicum
Chrysanthemum x morifolium
Carthamus tinctorius (reversed)
Centaurea diffusa (reversed)
Cynara cardunculus var. scolymu
Leontopodium leiolepis
Lactuca sativa (reversed)
Lactuca sativa var. salinas
Taraxacum amplum (A978)
Taraxacum obtusifrons (O978)
Taraxacum stridulum (S3)
Nicotiana tabacum
ndhD-ccsA
Aster spathulifolius
Jacobaea vulgaris
Leontopodium leiolepis
Chrysanthemum indicum
Chrysanthemum x morifolium
Artemisia montana
Artemisia frigida
Ageratina adenophora
Praxelis clematidea
Guizotia abyssinica
Parthenium argentatum
Helianthus annuus
Lasthenia burkei
Carthamus tinctorius
Centaurea diffusa
Cynara cardunculus
Taraxacum amplum (A978)
Taraxacum obtusifrons (O97
Taraxacum stridulum (S3)
Lactuca sativa
Lactuca sativa var.Salinas
Nicotiana tabacum
5'rps16-trnQ

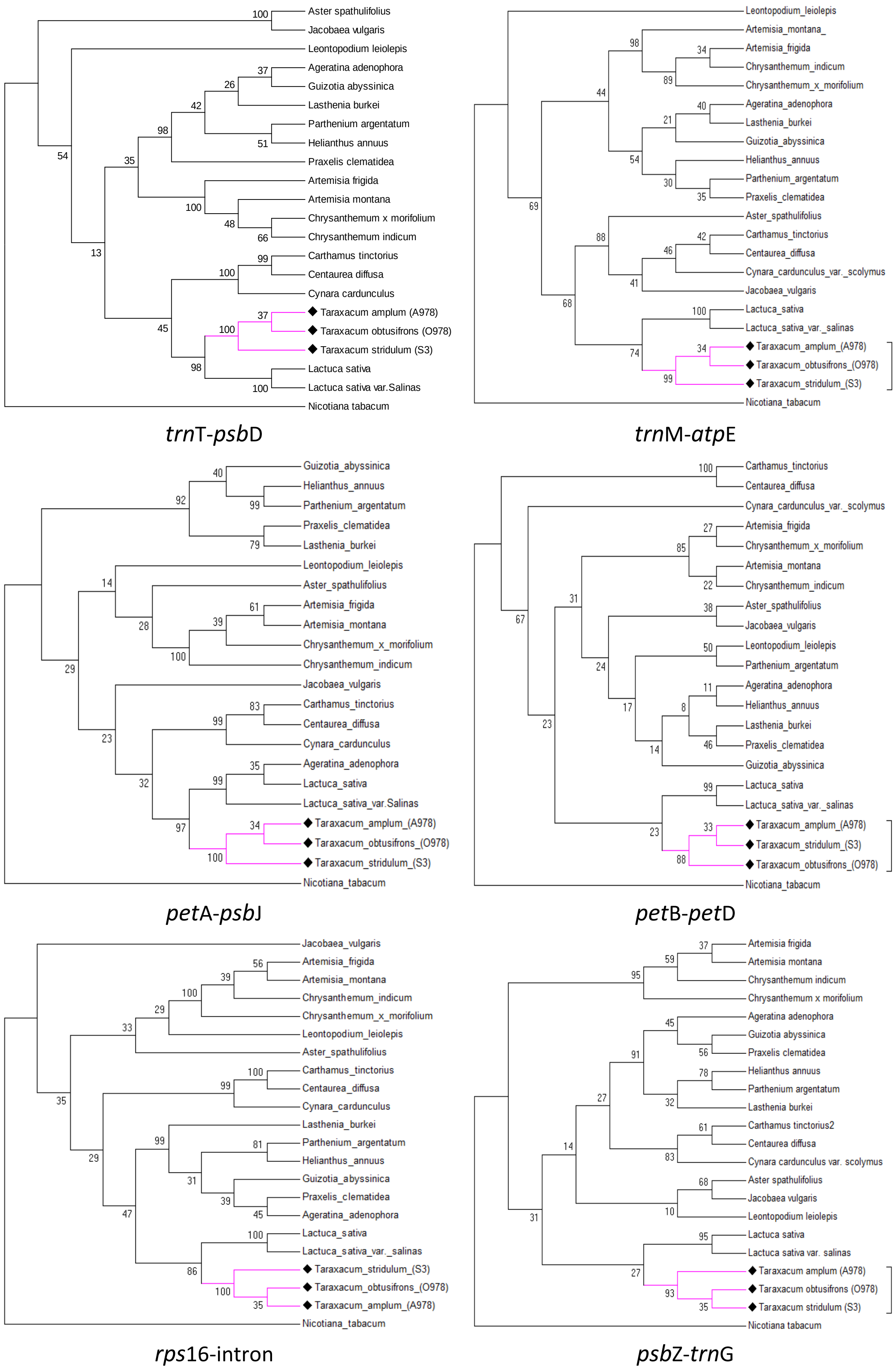

Aster spathulifolius
Jacobaea vulgaris
Leontopodium leiolepis
Ageratina adenophora
Guizotia abyssinica
Lasthenia burkei
Parthenium argentatum
Helianthus annuus
Praxelis clematidea
Artemisia frigida
Artemisia montana
Chrysanthemum x morifolium
Chrysanthemum indicum
Carthamus tinctorius
Centaurea diffusa
Cynara carduncculus
Taraxacum amplum (A978)
Taraxacum obtusifrons (O978)
Taraxacum stridulum (S3)
Lactuca sativa
Lactuca sativa var.Salinas
Nicotiana tabacum
trnT-psbD
Leontopodium_leiolepis
Artemisia_montana_
Artemisia_frigida
Chrysanthemum_indicum
Chrysanthemum_x_morifolium
Ageratina_adenophora
Lasthenia_burkei
Guizotia_abyssinica
Helianthus_annuus
Parthenium_argentatum
Praxelis_clematidea
Aster_spathulifolius
Carthamus_tinctorius
Centaurea_diffusa
Cynara_cardunculus_var._scolymus
Jacobaea_vulgaris
Lactuca_sativa
Lactuca_sativa_var._salinas
Taraxacum_amplum_(A978)
Taraxacum_obtusifrons_(O978)
Taraxacum_stridulum_(S3)
Nicotiana_tabacum
trnM-atpE
Guizotia_abyssinica
Helianthus_annuus
Parthenium_argentatum
Praxelis_clematidea
Lasthenia_burkei
Leontopodium_leiolepis
Aster_spathulifolius
Artemisia_frigida
Artemisia_montana
Chrysanthemum_x_morifolium
Chrysanthemum_indicum
Jacobaea_vulgaris
Carthamus_tinctorius
Centaurea_diffusa
Cynara_cardunculus
Ageratina_adenophora
Lactuca_sativa
Lactuca_sativa_var.Salinas
Taraxacum_amplum_(A978)
Taraxacum_obtusifrons_(O978)
Taraxacum_stridulum_(S3)
Nicotiana_tabacum
petA-psbJ
Carthamus_tinctorius
Centaurea_diffusa
Cynara_cardunculus_var._scolymus
Artemisia_frigida
Chrysanthemum_x_morifolium
Artemisia_montana
Chrysanthemum_indicum
Aster_spathulifolius
Jacobaea_vulgaris
Leontopodium_leiolepis
Parthenium_argentatum
Ageratina_adenophora
Helianthus_annuus
Lasthenia_burkei
Praxelis_clematidea
Guizotia_abyssinica
Lactuca_sativa
Lactuca_sativa_var._salinas
Taraxacum_amplum_(A978)
Taraxacum_stridulum_(S3)
Taraxacum_obtusifrons_(O978)
Nicotiana_tabacum
petB-petD
Jacobaea_vulgaris
Artemisia_frigida
Artemisia_montana
Chrysanthemum_indicum
Chrysanthemum_x_morifolium
Leontopodium_leiolepis
Aster_spathulifolius
Carthamus_tinctorius
Centaurea_diffusa
Cynara_carduncculus
Lasthenia_burkei
Parthenium_argentatum
Helianthus_annuus
Guizotia_abyssinica
Praxelis_clematidea
Ageratina_adenophora
Lactuca_sativa
Lactuca_sativa_var._salinas
Taraxacum_stridulum_(S3)
Taraxacum_obtusifrons_(O978)
Taraxacum_amplum_(A978)
Nicotiana_tabacum
rps16-intron
Artemisia frigida
Artemisia montana
Chrysanthemum indicum
Chrysanthemum x morifolium
Ageratina adenophora
Guizotia abyssinica
Praxelis clematidea
Helianthus annuus
Parthenium argentatum
Lasthenia burkei
Carthamus tinctorius2
Centaurea diffusa
Cynara cardunculus var. scolymus
Aster spathulifolius
Jacobaea vulgaris
Leontopodium leiolepis
Lactuca sativa
Lactuca sativa var. salinas
Taraxacum amplum (A978)
Taraxacum obtusifrons (O978)
Taraxacum stridulum (S3)
Nicotiana tabacum
psbZ-trnG

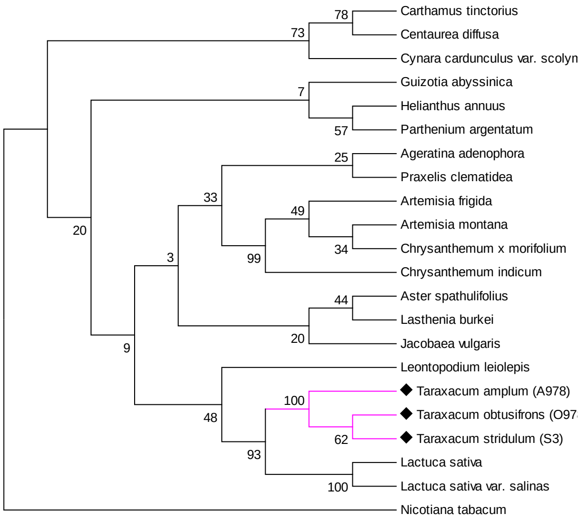

*trn*F-*ndh*J

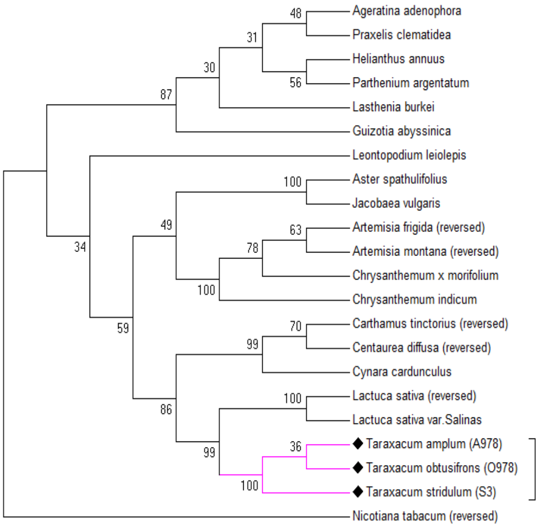

*rpl*32-*trn*L-UAG

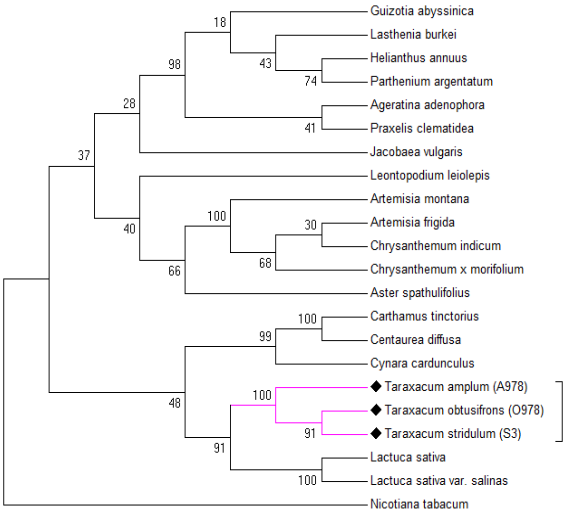

*psb*B

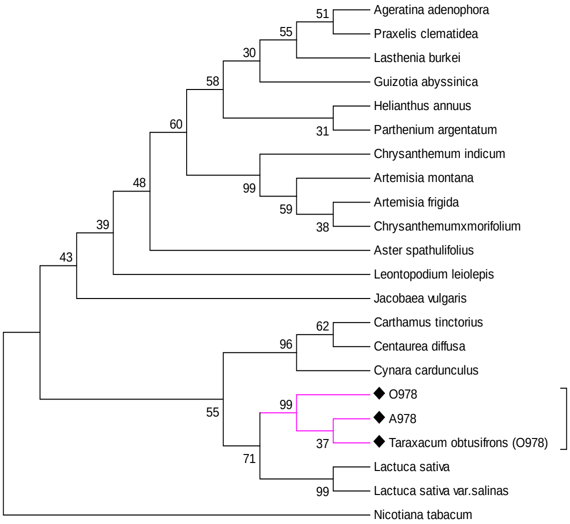

*trn*L

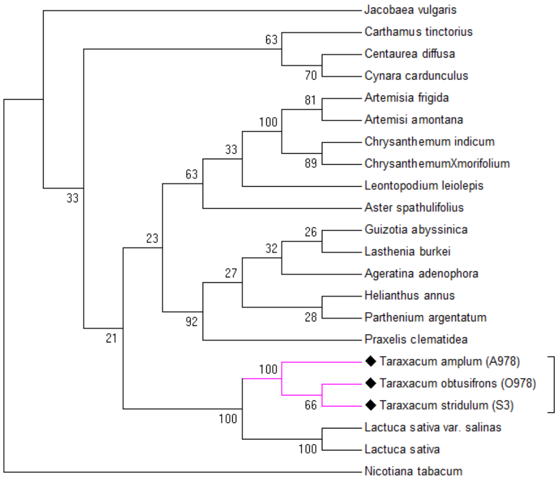

*acc*D

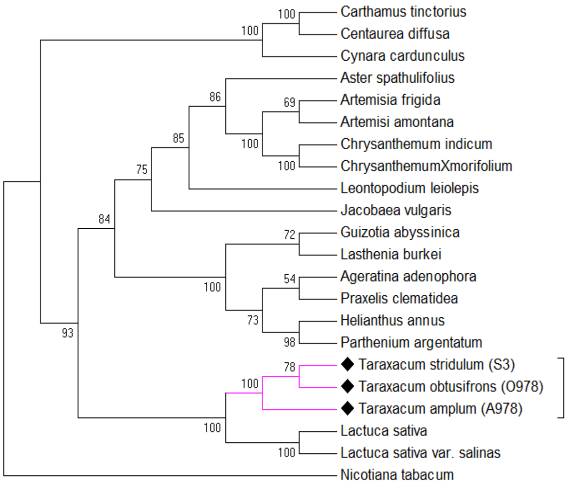

*rpo*C2

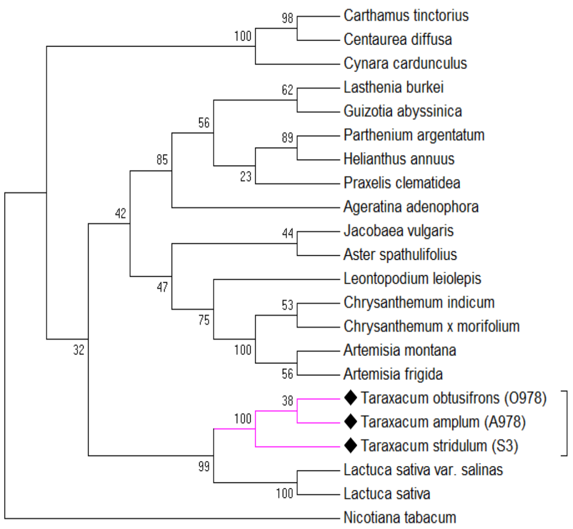

*rpo*C1

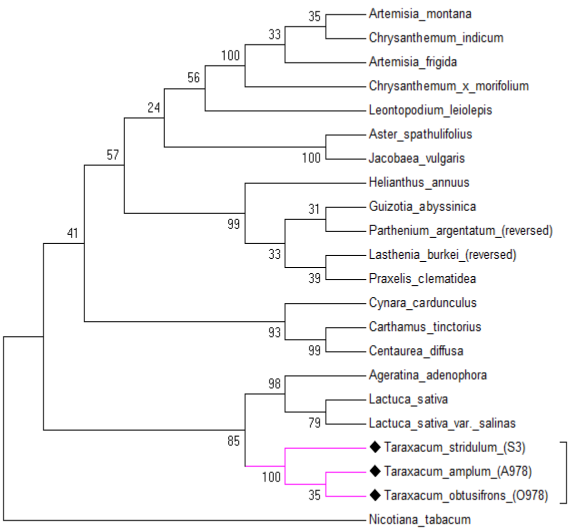

*clp*P

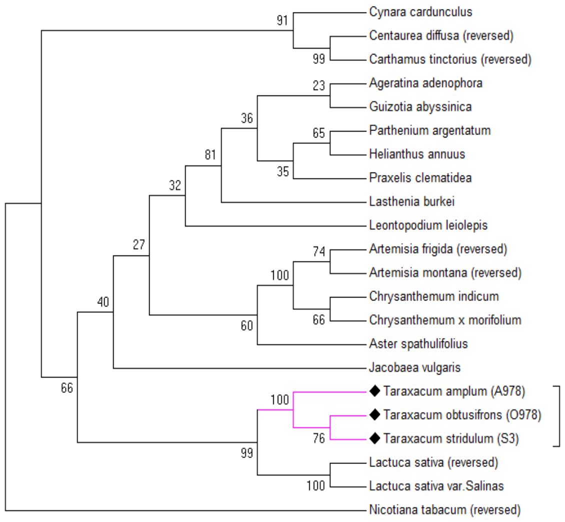

*ndh*D

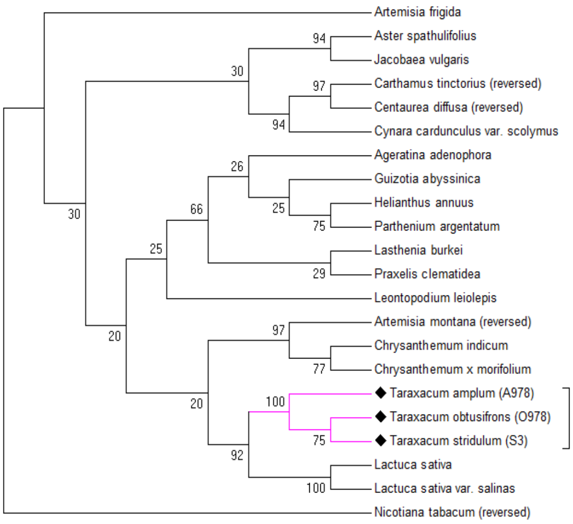

*ycf*1

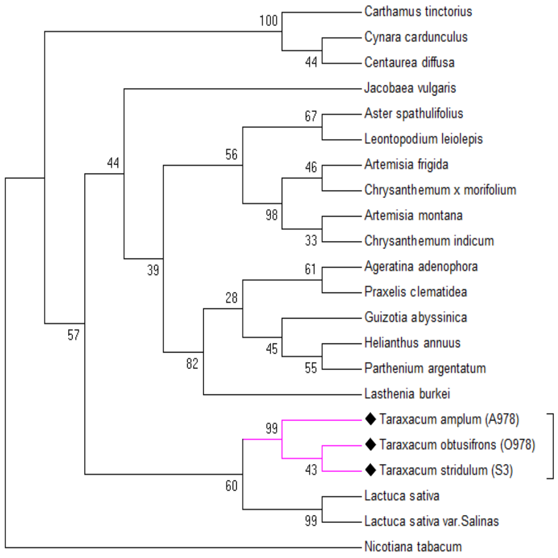

*rpl*22

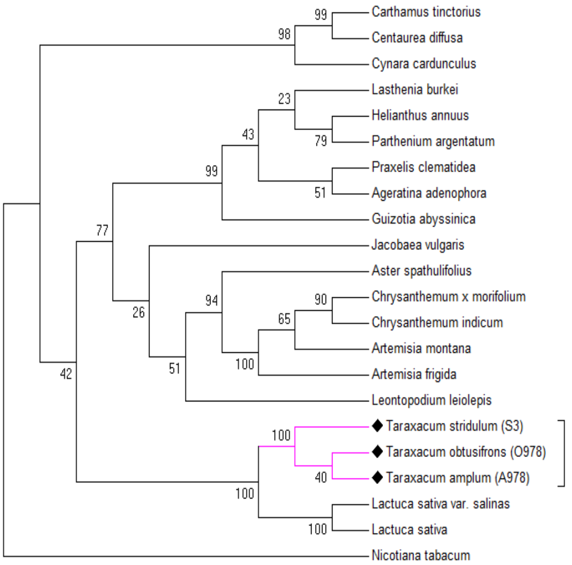

*ndh*F
